# Hydrogel adhesives for upper gastrointestinal wound healing

**DOI:** 10.7150/thno.124843

**Published:** 2026-01-01

**Authors:** Lianjun Ma, Fangming Gu, Jiaqian Hu, Xin Wang, Ying-Wei Yang

**Affiliations:** 1 China-Japan Union Hospital of Jilin University, Jilin University, Changchun 130033, P. R. China.; 2 International Joint Research Laboratory of Nano-Micro Architecture Chemistry, College of Chemistry, Jilin University, 2699 Qianjin Street, Changchun 130012, P. R. China.

**Keywords:** hydrogel, wound healing, hemostasis, combination therapy, gastrointestinal tract

## Abstract

This review discusses the latest progress in hydrogel adhesives for upper gastrointestinal wound healing. The specific acidic environment of the upper gastrointestinal tract makes treating upper gastrointestinal wounds, especially gastric bleeding and perforation, quite challenging. Recent research on hydrogels in biomedical applications has demonstrated immense potential, particularly in wound dressing. With three-dimensional network structures, hydrogels provide an efficient and safe strategy for hemostasis and wound repair in the digestive tract. Moreover, hydrogels possess excellent properties, including biocompatibility, biodegradability, mechanical strength, cell/tissue adhesion, and controllable release, making them suitable for complex wound-healing processes. This review summarizes the latest advancements in hydrogels for various upper gastrointestinal conditions, including esophageal stenosis, gastric ulcers, gastric bleeding, gastric perforation, and endoscopic submucosal dissection. We also highlight the merits and demerits of current hydrogel dressings to provide a deep understanding of their working mechanisms. Finally, the challenges and prospects of hydrogels for upper gastrointestinal applications are reviewed, aiming to inspire researchers in different fields and encourage the development of multifunctional hydrogels for effective upper gastrointestinal wound healing.

## 1. Introduction

The gastrointestinal (GI) tract, comprising the esophagus, stomach, small intestine (duodenum, jejunum, and ileum), and large intestine (cecum, colon, and rectum), plays a crucial role in the transportation, digestion, and absorption of food 1. GI wounds, such as esophageal stenosis, gastric hemorrhage, gastric perforation, and peptic ulcers, pose complex treatment challenges due to the unique physiological characteristics and dynamic microenvironment of the GI tract, particularly the presence of gastric acid. Frequent rebleeding, perforation, and infection remain the most difficult and prevalent clinical challenges. Although traditional treatment strategies, including suturing, clipping, and conventional hemostatic agents, have played an essential role in clinical practice, they often fail to provide prolonged adhesion and prevent healing in the acidic, dynamic gastric environment. Due to their strong wet adhesion and biocompatibility, hydrogel adhesives have emerged as promising candidates for GI wound management. However, conventional dressings are often inadequate or intolerable for wound sites, resulting in impaired wound healing 2. When gastric perforation occurs, stomach acid, partially digested food, and bacteria can enter the abdominal cavity, significantly increasing the risk of infection and severe complications 3. Therefore, innovative multifunctional dressings have been developed to repair, regenerate, or enhance dysfunctional organs or tissues in the GI tract 4, 5.

Given that strong interfacial adhesion to tissues is recognized as a critical factor influencing the overall robustness and reliability of therapeutic interventions, hydrogels have emerged as a key bridging material for the construction of adhesive dressings in the biomedical field. Hydrogels, built with a macromolecular backbone and side chains containing abundant reversible bonds, possess a typical three-dimensional (3D) network that can retain abundant water while exhibiting negligible changes in macroscopic solid-like rheological and adhesion behavior 6-8. Compared with conventional dressings, hydrogels can adhere more readily to biological tissues due to their similarity to human tissues and organs, coupled with their remarkable toughness and stretchability 9. In addition, hydrogels can prevent bacterial infections as physical barriers and concentrated platelets and coagulation factors by absorbing water, thereby shortening clotting time and accelerating the healing process 10. Recently, hydrogels have also been used to load and deliver other therapeutic agents, enabling the achievement of additional functions, some of which are particularly required in many biomedical applications, such as gelatin hemostatic sponge and fibrin glue 11.

In particular, the introduction of non-covalent supramolecular interactions, including van der Waals forces, hydrogen bonding, hydrophobic interactions, and electrostatic interactions, provides a facile and effective method to endow hydrogels with reversible properties, such as degradability, self-healing, and stimuli-responsive abilities. These intriguing characteristics position hydrogels as promising materials for assisting hemostasis and wound healing in the GI tract 11. Over the past few decades, significant advances have occurred in the study of viscous hydrogels, with substantial progress in their structural design and biomedical applications. For instance, Artzi *et al.* 12 reported a sprayable hydrogel that facilitates continuous adhesion to GI lesions, enabling the biomaterial to instantly gel and adhere to tissues, thereby forming a protective barrier. Gastric acid, with a pH<1.5, is an intractable obstacle to gastric wound therapy. Most drugs and conventional hemostatic dressings are inevitably damaged by the highly acidic microenvironment, leading to severely weakened adhesiveness. Recently, acid-resistant hydrogels have been developed and proven to be a boon to these patients. For example, Liu *et al.* 13 introduced an acid-resistant hydrogel bioadhesive (AtGel) comprising an acid-resistant hydrogel substrate and an adhesive polymer brush layer. The hydrogel demonstrates effective acid resistance and maintains strong interfacial toughness and robust bioadhesion, even after immersion in a simulated gastric fluid medium for 240 hours.

Although the research progress of hydrogels has been summarized in several interesting reviews, for instance, the research status of self-healing hydrogels in wound management 11, the applications of antioxidant hydrogels in oxidative stress-related diseases 14, and the application of polysaccharide-based hydrogels in different types of GI wounds 15, a thorough review entirely focusing on the hydrogels for upper GI tissue engineering applications has not been found in the literature, especially given that various hydrogels have emerged in the last five years. Existing clinical treatments for upper GI wound healing include suturing, clips, stents, and fibrin glue. These methods often face challenges, including complexity, patient discomfort, and an increased risk of infection or recurrence. Hydrogel adhesives offer advantages over traditional methods, such as strong wet adhesion, acid resistance, controllable degradation, injectability, and localized drug delivery. However, potential disadvantages, such as cost and durability, remain compared with conventional treatment approaches.

**Scheme 1:**
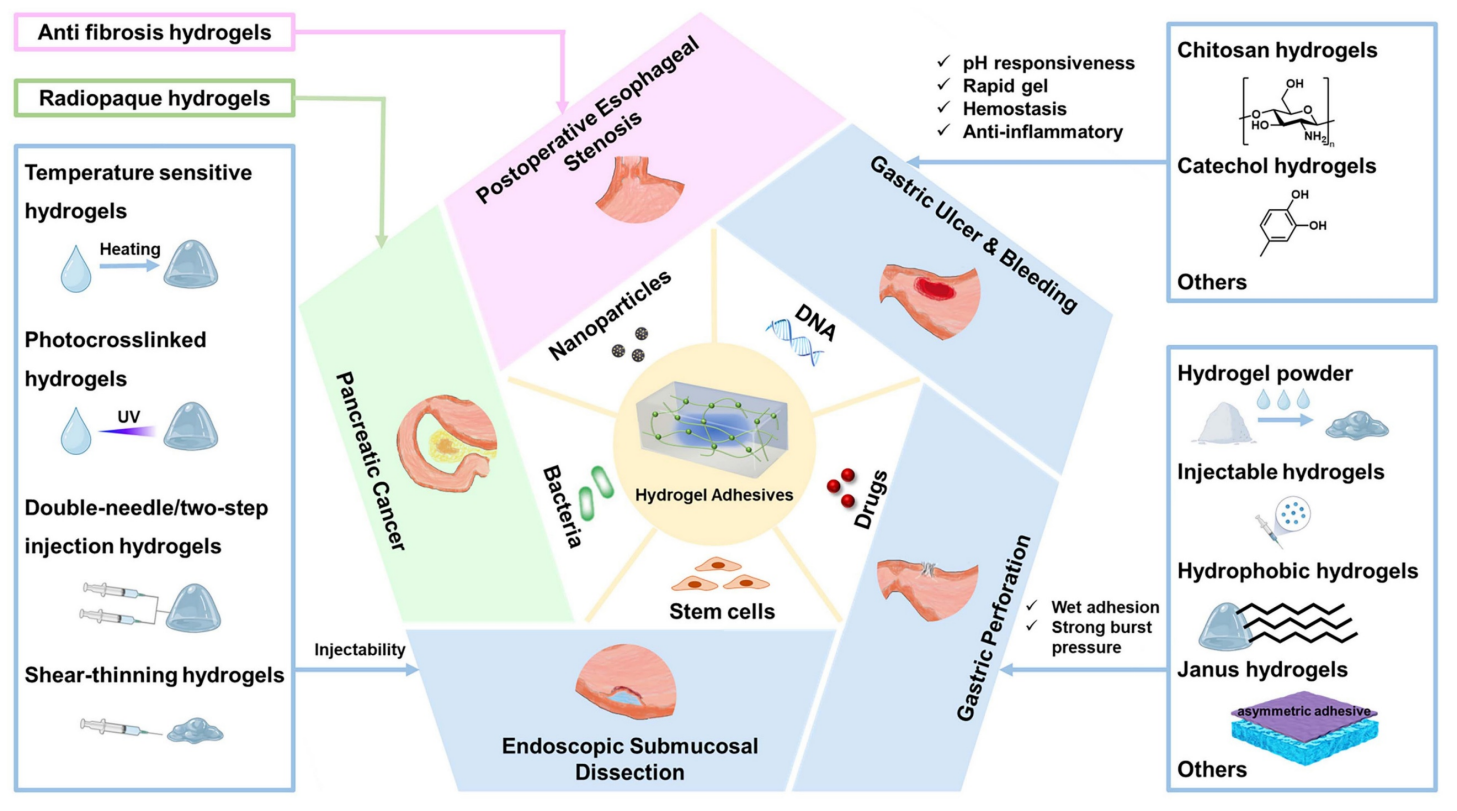
The functional classification and specific applications of hydrogels, showing the types and characteristics of hydrogels used for postoperative esophageal stenosis, gastric ulcers and bleeding, gastric perforation, endoscopic submucosal dissection (ESD), and pancreatic cancer.

In this review, we aim to advance the development of hydrogel adhesives in the treatment and management of upper GI tissue injuries. First, we discussed the classification of advanced hydrogels by function, with an emphasis on their design, synthesis, and potential applications (Scheme 1). Then, we systematically summarized the applications of various advanced hydrogels in common GI diseases associated with tissue damage and their corresponding microenvironmental characteristics, including postoperative esophageal stenosis, gastric ulcer and bleeding, gastric perforation, endoscopic surgery incision, and pancreatic cancer (Scheme 1, Tables 1-3). Finally, the challenges and prospects in this research field are discussed from both fundamental research and translational medicine perspectives.

## 2. Classification of hydrogels

Hydrogel synthesis and structural design directly determine their properties and clinical applications, for example, acid-resistant groups and dense crosslinking enhance gastric stability, while thermosensitivity, shear-thinning properties, or rapid photocrosslinking enable injectability or sprayability for endoscopic delivery.

### 2.1 Classification of hydrogels by cross-linking methods

Hydrogels can be categorized based on the interactions between polymer chains into three types: chemical hydrogels, physical hydrogels, and hybrid hydrogels. Non-covalent physical crosslinking methods, such as van der Waals forces, hydrophobic interactions, and electrostatic interactions, endow hydrogels with reversibility and self-healing, but exhibit limited structural stability 16. These physically crosslinked hydrogels are prone to degradation under gastric acid, restricting their durability in GI environments.

In contrast, chemical crosslinked hydrogels, constructed through radical polymerization, interpenetrating polymer networks, radiation polymerization, and complementary groups, exhibit greater mechanical strength and environmental stability. However, their slow degradation rates may raise long-term safety concerns, warranting further optimization 17. Hybrid crosslinking strategies that integrate physical and chemical methods offer a rational design approach. Such hybrid hydrogels combine reversibility and stability, resulting in improved performance in complex gastrointestinal environments and providing meaningful insights for the design of advanced hydrogels.

### 2.2 Classification of hydrogels by components

Hydrogels can be divided into simple hydrogels and hybrid hydrogels based on their components. Generally, simple hydrogels possess only a porous skeleton structure, providing only basic adhesion and hydration, while their therapeutic functionality is often limited. In contrast, hybrid hydrogels incorporate nanoparticles, peptides, DNA, drugs, cells, or bacteria, endowing them with enhanced mechanical robustness and multifunctional therapeutic potential.

#### 2.2.1 Simple hydrogels

Simple hydrogels are easier to prepare. They not only have their own excellent adhesion and mechanical properties, but also can be used as a skeleton and carrier to participate in the composition of other materials. For instance, Xie *et al.* 18 introduced a simple hydrogel composed of carboxymethyl chitosan (CMCS), sodium alginate (SA), and oxidized dextran (ODE), which can achieve rapid hemostasis by adhering to the injured tissue and prevent infection induced by *S. aureus* due to the antibacterial effect of CMCS. Furthermore, hydrogels can mimic the 3D structure of the extracellular matrix and accelerate tissue repair in mice.

#### 2.2.2 Hybrid hydrogels

**Figure 1:**
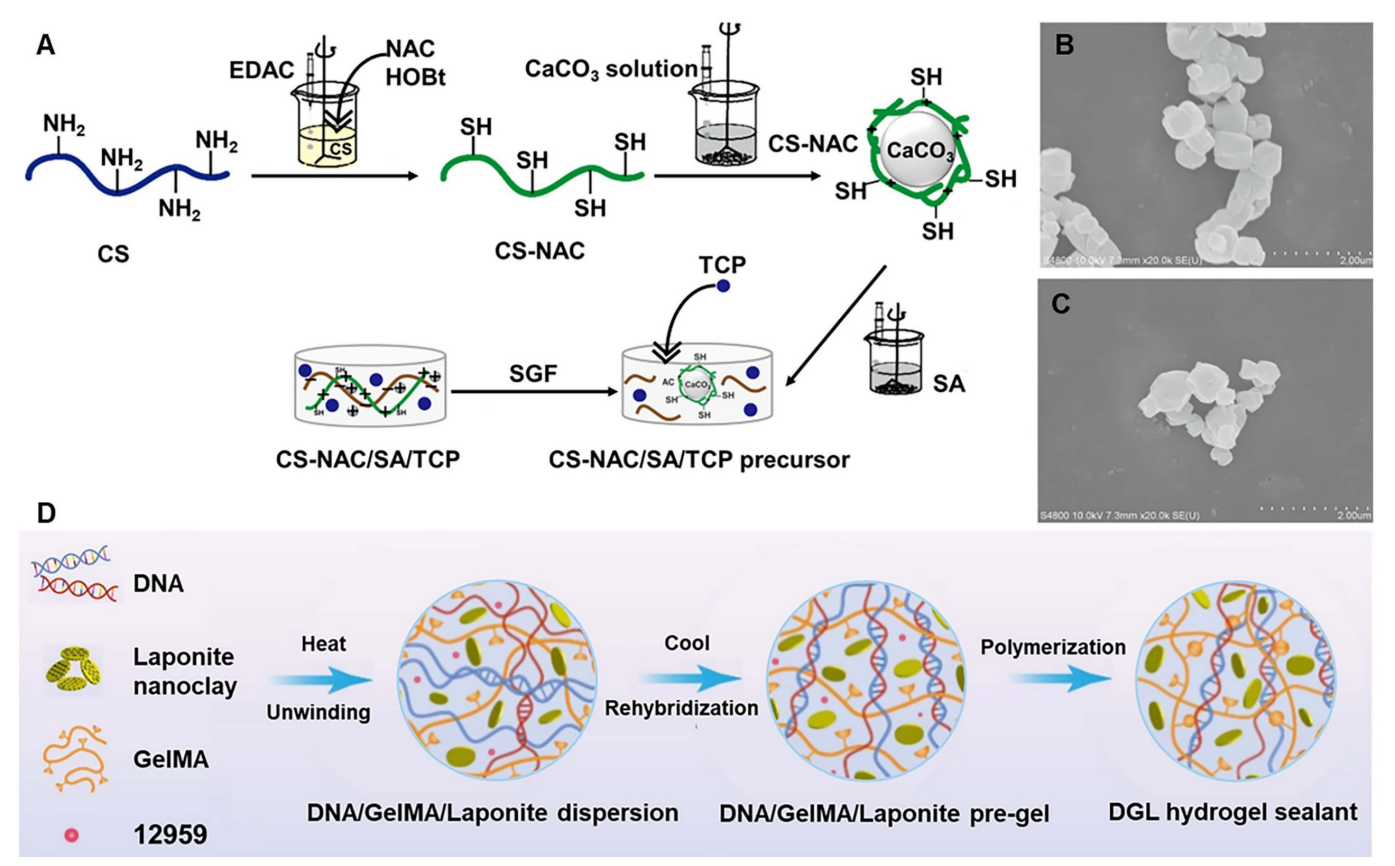
Hydrogels containing nanoparticles or DNA. (A) Schematic diagram displaying the synthesis of CS-NAC/SA/TCP hydrogel for alcohol-induced gastric mucosal injury. Adapted with permission from 19, copyright 2022, Elsevier. (B) SEM of pure nano-CaCO_3_ particles. (C) SEM of CS-NAC (CaCO_3_) particles. (D) Schematic diagram showing the design and preparation of DGL sealant hydrogel through highly base complementary pairing of DNA. Adapted with permission from 21, copyright 2023, Elsevier.

***Hydrogels with nanoparticles.*** Recently, with the rapid development of nanotechnology, the potential applications of nanomaterials in medicine have advanced significantly. Hybrid hydrogels with diverse functions can be developed by incorporating nanoparticles into the hydrogel matrix. Lu *et al.* 19 introduced a pH-sensitive hydrogel comprising chitosan (CS), SA, and tilapia collagen peptide to protect against gastric mucosal injury induced by alcohol, demonstrating continuous release of TCP, strong mucosal adhesion, and superior biodegradability (Figure 1A). The hydrogel used CS-NAC-encapsulating CaCO_3_ nanoparticles to achieve gastric acid-responsiveness. The SEM image of pure nano-CaCO_3_ particles was almost cubic (Figure 1B), while CS-NAC (CaCO_3_) particles were almost circular (Figure 1C). When gastric acid gradually decomposes CaCO_3_, releasing Ca^2+^, Ca^2+^ and SA form a classic eggshell structure, indicating the formation of a hydrogel. Moreover, Chen *et al.* 20 loaded hydrogels with nanoparticles with magneto-thermal function and promoted the gel-sol transition of hydrogels via magneto-thermal treatment to fill gastric ulcers.

***Hydrogels with deoxyribonucleic acid (DNA).*** Bases are essential components of genetic material, forming stable structures of DNA and ribonucleic acid (RNA) through hydrogen bonding between purines and pyrimidines. Due to their precise base-pair recognition, DNA macromolecules can be used to construct dynamic hydrogel materials. For example, Xie *et al.* 21 applied gelatin, Laponite nanoclay, and DNA to build a nano-enabled DNA supramolecular hydrogel sealant (DGL) (Figure 1D), which exhibits excellent tissue adhesion, injectability, and self-healing properties, and can rapidly stop bleeding and prevent postoperative adhesions. DNA with highly complementary base pairing is the primary building block for constructing a physical interconnection network, enabling the DGL hydrogel to be dynamically reversible through multiple hydrogen bonds.

***Hydrogels with drugs.*** Hydrogels can realize controlled and continuous delivery of various drugs. For example, Dave *et al.* 22 added paclitaxel and 6-aminocaproic acid to a self-assembled small molecule hydrogel based on diglycerol monostearate for anticancer and hemostatic effects, which slowly degraded in the presence of lipase. Parsa *et al.* 23 added tetracycline to a CS hydrogel to construct a hybrid hydrogel suitable for wound dressings and drug delivery. The hydrogel slowly released a therapeutic dose of tetracycline within five days. In addition, modifications to drug molecules can contribute to the formation of a hydrogel crosslinking network. For example, Ouyang *et al.* 14 used amino-modified montmorillonite to form amide linkages with the carboxyl of CMCS and oxidized sodium alginate (OSA), so that montmorillonite was evenly dispersed in the hydrogel.

***Hydrogels with cells.*** Adult stem cells, such as endothelial progenitor cells, mesenchymal matrix stem cells, and adipose tissue-derived stem cells, have been used in applications for injured tissue healing 24. Due to the swallowing and wriggling of food in the GI tract, stem cells used for treatment will be lost in the early stages of treatment. This problem can be solved by loading stem cells into hydrogels. For example, Chung *et al.* 25 loaded fat-derived stem cells into a hydrogel to prepare a hybrid hydrogel for postoperative esophageal stricture, thereby regulating the regenerative process through the paracrine effects of stem cells. In addition to stem cells, extracellular vesicles also contribute to wound healing and regulate homeostasis through cell recruitment, proliferation, migration, and differentiation 26.

***Hydrogels with bacteria.*** Probiotic therapy can also be used for bacterial interference and immune regulation 27. Applying hydrogel to the base of probiotic therapy can effectively prevent the escape of probiotics; for example, Ming *et al.* 28 wrapped Lactobacillus reuteri into hydrogel microspheres via emulsion polymerization and formed a hydrogel at the wound site under light irradiation through covalent crosslinking of materials. Lactobacillus reuteri can decrease local pH and produce antibacterial agents to repress the growth of pathogenic bacteria. The experiments showed that the wound treated by live bacterial hydrogel had a lighter inflammatory response and faster healing. However, this technology has not yet been applied to the GI tract.

Overall, chemical bonding and physical interactions synergistically influence the adhesion between hydrogels and GI tissues. Catechol hydrogel adhesives usually interact with various tissue components, such as mucin and collagen, through covalent bonds or coordination, generally possessing good acid resistance, durability, and mechanical stability. Schiff-base hydrogels are dynamically covalently bonded and exhibit moderate stability under mild acidic conditions. Non-covalent supramolecular interactions, including hydrogen bonding, electrostatic interactions, hydrophobic anchoring, and van der Waals forces, promote rapid initial adhesion. However, due to their susceptibility to microenvironmental fluctuations, supramolecular hydrogels are better suited for short-term adhesion.

### 2.3 Design principles for acid-resistant and tissue-adhesive hydrogels

The adhesion and stability of hydrogel adhesives are usually affected by multiple covalent and non-covalent interactions. Functional groups, spatial architecture, and incorporated components directly determine their adhesion durability and anti-swelling capacity in highly acidic, enzyme-rich, and peristaltic environments. Covalent interactions form the primary network of hydrogel adhesives, such as catechol coupling, thiourea-catechol protection, Schiff-base linkages, and photo-crosslinking 29, 30. These interactions can resist acid hydrolysis and prevent premature degradation. Dynamic interactions, including imine exchange and boronate ester linkages, can repeatedly break and reform in response to acidic fluctuations and peristaltic stress, endowing hydrogels with self-repairing and adaptive abilities 31.

Non-covalent interactions act as secondary modulators to further reinforce adhesion of hydrogels, including hydrogen bonding, hydrophobic anchoring, cation-π interactions, and electrostatic bridging. Their hydrophobic groups can expel water molecules from the wound site, promoting the hydrogel's close adherence to the tissue and reducing acid penetration 32. In addition, hydrogels can bind to gastric mucins or ECM residues through electrostatic interactions 33. The combined contribution of these reversible interactions significantly enhances the wet adhesion performance on gastric mucosa.

The network architecture and functional fillers of hydrogel adhesives can reduce acid-induced swelling, delay pepsin infiltration, and enhance stability under gastric peristalsis. In particular, the chemical-physical cross-linked network, which integrates permanent bonds and sacrificial bonds, exhibits excellent self-repairing capability 21. Moreover, the incorporation of functional components such as DNA, peptides, polysaccharides, or nanomaterials can also regulate the adhesion, rheology, and degradation properties of hydrogels. In summary, the acid resistance, tissue adhesion, and dynamic stability of hydrogel adhesives suitable for the upper GI environment depend on the synergistic contribution of multiple interactions. Rational design and regulation of these interactions will yield effective strategies for developing next-generation hydrogel adhesives.

## 3. Applications of hydrogels

Selecting appropriate hydrogels based on clinical needs is crucial for treatment. Generally, injectable, sprayable, and photo-crosslinked hydrogels are mainly applied in endoscopic procedures, such as hemostasis, ulcer repair, and submucosal injection during ESD, due to their excellent *in situ* gelation and controlled fluidity. In contrast, prefabricated, mechanically reinforced, or powder-type hydrogels are mainly suitable for open surgical repairs because they require durable mechanical support. Therefore, we highlight recent advances in the clinical applications of hydrogels for various gastric diseases. Moreover, corresponding key performance parameters of hydrogel adhesives for different gastric disease models were summarized in Table 4.

### 3.1 Postoperative esophageal stricture

Esophageal cancer (EC) is the seventh leading cause of cancer death in the world 34. Traditional endoscopic approaches, such as repeated dilation and stenting, are limited by mechanical trauma and insufficient promotion of tissue repair or epithelial regeneration. Hydrogel adhesives, particularly when combined with thermoresponsive or drug-loading properties, provide sustained support and promote epithelial regeneration. Nonetheless, challenges such as long-term biostability and controlled degradation remain to be addressed 35. Nevertheless, postoperative stenosis is a common perioperative complication in endoscopic submucosal dissection (ESD) of the esophagus, with a particularly high incidence when the mucosal resection reaches three-quarters of the esophageal circumference 36-41.

Recently, several new biomedical materials have emerged for preventing esophageal stenosis. Lee and Park developed a hydrogel-impregnated robust interlocking nano connector (HiRINC) on a self-expandable metallic stent to connect the hydrogel and the extra-luminal stent strut surface. HiRINC has increased bonding sites and enhances mechanical interlocking and hydrogel bonding with hydrogel, thanks to its network-like porous structure. In the rat and porcine esophagus, HiRINC remains long-term anchored at the target esophageal stricture area by absorbing and distributing mechanical forces during peristalsis, offering new avenues to achieving a durable anti-restenosis approach.

A variety of methods have been investigated to prevent stenosis after ESD, including physical dilation, glucocorticoid application, anti-inflammatory or anti-fibrotic drug use, and autologous tissue transplantation 42. The preponderance of evidence suggests that corticosteroid therapy reduces the incidence of postoperative esophageal stenosis. Recently, advances in biomedical materials research have led to several new methods for preventing esophageal stenosis. For example, Coffin *et al.* 26 combined extracellular vesicles (EV) of stromal cells with a temperature-responsive hydrogel to prevent stenosis occurrence after endoscopic surgery in pig models. The experimental results confirmed that the rate of esophageal stenosis was markedly reduced, the average fibrosis area was reduced, and the regenerated mucosal muscle layer was larger in the EV+gel group. Chung *et al.* 25 designed a cell transplantation platform based on an ascidian-inspired hyaluronic acid hydrogel loaded with adipose-derived stem cells to alleviate esophageal stenosis. Excessive fibroblast differentiation and long-term inflammation are important factors in the development of esophageal stenosis; inhibiting fibroblast proliferation and accelerating early wound healing can effectively prevent its development 43. Wang *et al.* 44 reported a thermosensitive hydrogel containing CS for triamcinolone delivery, which can be injected into esophageal wounds. Pan *et al.* 45 reported a double-crosslinked hydrogel containing SA, gelatin powder, aqueous transglutaminase, and Ca^2+^ to prevent esophageal stenosis after ESD. Effective delivery of dressings to the ESD wound site is also important. It is also crucial to update the structure of the endoscope to adapt to the treatment methods of injection or spraying drugs. Fujiyabu *et al.* 46 combined polytetrafluoroethylene tubes with five different structures of catheter tip nozzles manufactured using 3D printers to develop a powder applicator for endoscopic powder delivery, achieving lateral powder delivery at the pointed end of endoscopic catheters, thereby enabling effective and efficient delivery.

### 3.2 Gastric diseases

Gastric diseases, such as gastric ulcer, gastric perforation, and endoscopic submucosal dissection (ESD), are often accompanied by symptoms such as mucosal erosion and bleeding, which may be caused by excessive acid, infection, or drug use. Conventional treatment methods such as proton pump inhibitors and endoscopic clipping provide temporary relief but often fail to achieve long-term mucosal protection, resulting in recurrent bleeding and delayed healing. Hydrogel adhesives exhibit strong wet adhesion, drug delivery capacity, and *in situ* protective barrier capability, providing a promising alternative for gastric ulcer treatment. However, long-term stability and controlled degradation in the dynamic gastric environment remain critical challenges.

#### 3.2.1 Gastric ulcer and bleeding

Gastrointestinal bleeding (GIB) is a medical emergency and the most common cause of hospitalization for digestive system diseases in the majority of countries 47. GIB can be caused by inflammation, mechanical damage, vascular disease, tumors, and other factors within the digestive tract itself, as well as by lesions in adjacent organs and systemic diseases affecting the digestive tract 48. Among these, peptic ulcers are the most common 49. Although the incidence of upper GIB has been declining worldwide, it still has an unnegligible high incidence and mortality rate 50. Endoscopy is the gold standard for GI hemostasis, and acute hemostasis can be achieved in most cases 51, 52. However, in certain exceptional cases, such as diffuse bleeding, bleeding in areas difficult to reach by endoscopy, tumor bleeding, and recurrent bleeding, the therapeutic effect of endoscopic hemostasis is limited 53. Therefore, it is very crucial to develop a safe, effective, and stable hemostatic agent.

**Figure 2:**
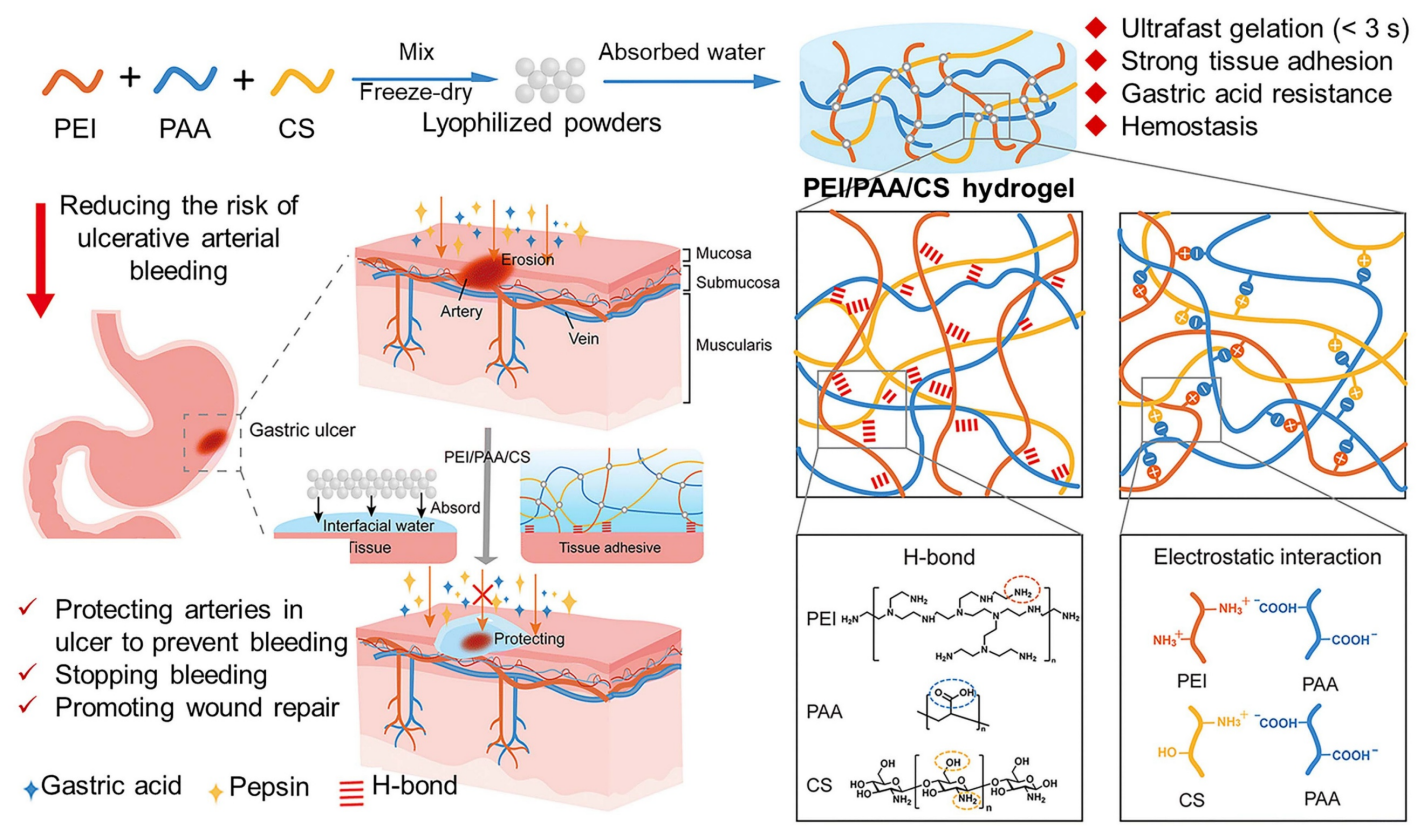
Schematic diagram showing the synthesis of PEI/PAA/CS hydrogel and the formation through hydrogen bonds and electrostatic interactions, as well as the adhesion mechanism of PEI/PAA/CS hydrogel in the gastric mucosa, which prevents bleeding and promotes wound repair. Adapted with permission from 53, copyright 2023, The Royal Society of Chemistry.

CS is composed of a copolymer of N-acetylglucosamine and glucosamine linked by β(1-4) 54, 55, first published by Schorigin and colleagues in 1935 56. Currently, hydrogels based on CS are widely used in multiple biomedical fields, including tissue engineering, controlled drug delivery, and wound repair. 57, 58. CS can also improve the biocompatibility of materials. Ni *et al.* 53 introduced CS into polyethyleneimine/polyacrylic acid (PEI/PAA) hydrogel and developed PEI/PAA/CS multi-functional hydrogel to reduce the risk of gastrorrhagia. The mixing of CS strengthened electrostatic interactions and hydrogen bonds in the original hydrogel, increased its crosslinking density, and enhanced its tissue adhesion. Meanwhile, due to the superior biocompatibility and hemostatic functions of CS, the capabilities of PEI/PAA/CS hydrogels have been improved. The powder of this hydrogel, which was produced by lyophilization and grinding, can absorb water from the surface of the tissue to quickly form a hydrogel. And it has strong tissue adhesion, which can adhere to the ulcer wound to avoid mucosal bleeding and promote tissue healing (Figure 2). In addition, incorporating CS into other hydrogels can also significantly enhance the material's water absorption capacity and accelerate blood coagulation, achieving effective hemostasis of gastric bleeding within 20 seconds 59.

**Figure 3:**
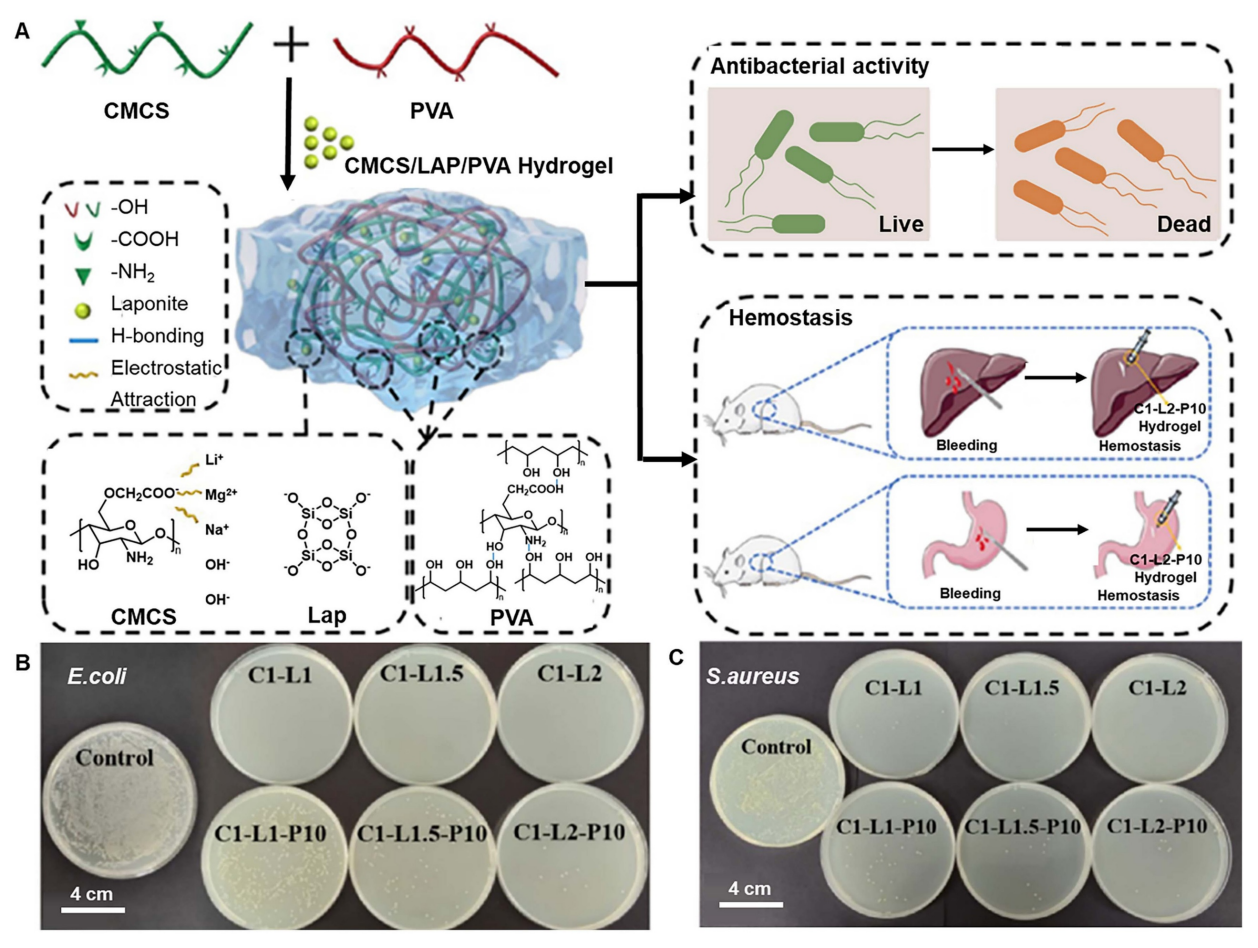
The antibacterial hydrogel based on CMCS for gastric ulcer and bleeding. (A) Schematic diagram displaying the synthesis of CMCS/LAP/PVA hydrogel cross-linking through electrostatic interactions and hydrogen bonds, and the hemostatic and antimicrobial properties of CMCS/LAP/PVA hydrogel. Colony growth of *E. coli*. (B) and *S. aureus* (C). Adapted with permission from 60, copyright 2023, American Chemical Society.

Compared with CS, CMCS has excellent hydrophilicity, appropriate viscosity, and injection pressure, and can combine with the bacterial cytoderm and destroy its structure, inducing bacterial death 60. Therefore, CMCS has been widely used as a hydrogel. CMCS/LAP/PVA hydrogel was formed via hydrogen bonds and electrostatic interactions between CMCS, lithium magnesium sodium salt (LAP), and poly(vinyl alcohol) (PVA), which has excellent hemostasis and antibacterial ability (Figure 3A) 60. It exhibited superior resistance against *S. aureus* and *E. coli* (Figure 3B-C). Other hydrogels based on CS also show excellent performance; for example, Liu *et al.* 61 introduced a chitosan hydrogel powder (HP) that forms hydrogels via electrostatic interactions within 5 seconds of contact with liquid. Washing with sodium bicarbonate solution can selectively remove the hydrogel.

**Figure 4:**
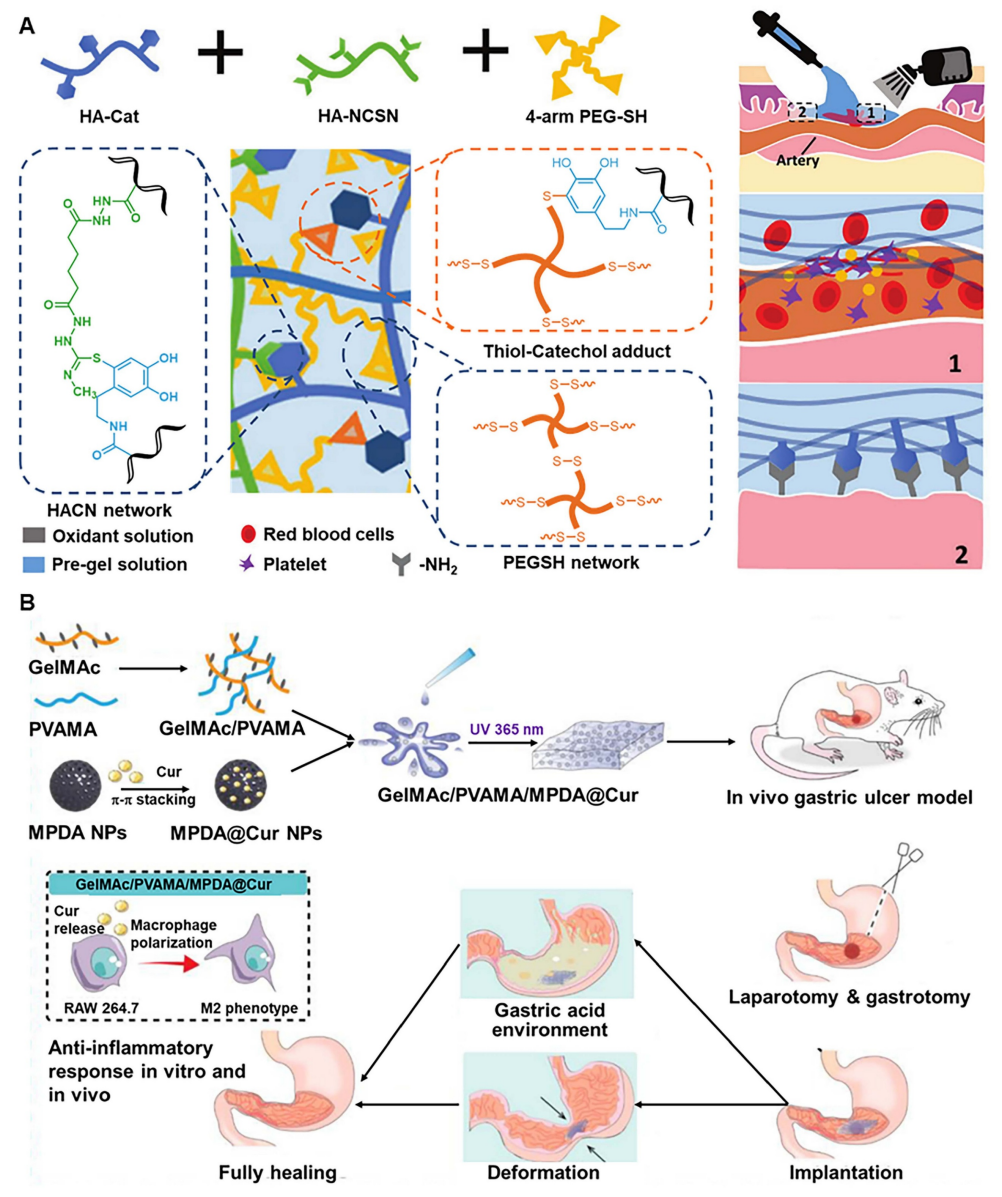
Catechol-related hydrogels for gastric ulcer and bleeding. (A) Schematic illustration showing the chemical structure, utilization process, the hemostasis and adhesion mechanisms of the HACN-PEG hydrogel. Adapted with permission from 29, copyright 2021, Wiley-VCH GmbH. (B) Schematic illustration showing the presentation of the GelMAc/PVAMA/MPDA@Cur hydrogel, which loads Cur into MPDA NPs through π-π stacking and hydrogen bonds for promoting gastric ulcer repair through regulating immune response. Adapted with permission from 69, copyright 2022, Elsevier.

Hydrogels based on thiourea-catechol reactions are widely used because of their strong mechanical properties and extremely short gel time 29, 62-64. In nature, mussels achieve strong underwater adhesion by secreting catechol-rich mussel adhesive protein 65. By mimicking mussel adhesive protein, many catechol-related (CAT-related) hydrogels exhibit strong adhesion to the surfaces of most objects 66-68. However, when CAT is exposed to oxidants (such as O_2_), it is inevitably oxidized to quinones, leading to loss of adhesion. As an excellent nucleophile, the thiourea group (NCSN) forms crosslinks with the conjugated catechol structure under acidic conditions to improve the preservation of the catechol structure, resulting in significantly enhanced adhesion 64. Xu *et al.* introduced a biomimetic hydrogel based on thiourea-CAT coupling, which showed ultrafast gelation independent of pH value 62. The hydrogel can be transported to the designated position via the endoscope, and a low-concentration oxidant can be sprayed onto the hydrogel precursor, which quickly forms a stable, viscous hydrogel *in situ* and remains in place for at least 48 hours. The introduction of the second crosslinked network can optimize the hemostatic application of this hydrogel. The research group introduced 4-arm thiolated polyethylene glycol (PEG) into HA-CAT-NCSN hydrogel to obtain a hybrid hydrogel (HACN-PEG hydrogel), which has stronger mechanical properties and shorter gelation time in relation to fibrin hydrogels or hydrogels with a single network (Figure 4A) 29. In addition, Zhang *et al.* mixed catechol motif-modified methacrylated gelatin (GelMAc) and polyvinyl methacrylate (PVAMA) and mesoporous polydopamine (MPDA) nanoparticles to form GelMAc/PVAMA/MPDA@Cur hydrogel, in which MPDA is used as a drug carrier and loaded with curcumin (Cur) through π-π stacking and hydrogen bonds (Figure 4B). This hydrogel was gelatinized under ultraviolet light and demonstrated unique stability in gastric tissue under acidic and oxidative stress conditions (H_2_O_2_). Additionally, it can promote macrophage polarization, thereby regulating inflammation and facilitating tissue repair 69.

Hydrogels based on some other materials also show excellent performance, for example, the hydrogels based on acryloyl showed superior injectability, self-healing ability, and stable adhesion behavior under stomach conditions, demonstrating their hemostasis function and promoting wound repair 70. However, its gelation time can reach 9 minutes, and the initial *in vitro* pre-polymerization step of 3 minutes increases the complexity of clinical procedures, thereby limiting its transformation potential. The polyurethane/small intestinal submucosa (PU/SIS) hydrogel has distinctive pH-sensitive properties 71. It can rapidly form a dense film in acidic conditions to protect GES-1 cells and accelerate ulcer healing. The hydrogel made of methacryloylhaluronic acid (MA-HA), 6-acrylamidohexanoic acid (AA), and ginsenoside Rg1 as raw materials is also pH-sensitive, which can shrink and tighten the wound in an acidic environment, while releasing Rg1 with anti-inflammatory effect 30. Artzi *et al.* 12 designed a sprayable bioadhesive hydrogel, GastroShield, which consists of two low-viscosity precursors. Due to the reduced viscosity of the precursors, their delivery will be more convenient via a catheter inserted into an endoscope channel. The precursor solution rapidly reacts upon contact with mucosal tissue, producing cohesive tissue shields. In most cases, the treatment of gastric ulcers and bleeding is guided by endoscopy. The injectability of hydrogel provides significant convenience for endoscopic therapy, which is the main future direction for hydrogel materials in the GI tract. However, keratin-based hydrogels can be administered orally without endoscopic guidance to form a high-viscosity gel at the surface of gastric wounds, especially at ulcer sites 72. This hydrogel can effectively improve the compliance in patients with mild gastric ulcers. Strong adhesion also plays a significant role in hemostasis. Ying *et al.* 73 reported an electroadhesive hydrogel for the treatment of recurrent GI bleeding. Under electrical stimulation, the hydrogel cationic polymer interacts with multiple proteins in deep mucosal tissue, increasing adhesion energy by 30 times. This technology allows the hydrogel to remain in the body for up to 30 days. Overall, the hemostatic performance of hydrogel adhesives used for gastric ulcers mainly depends on rapid interfacial water absorption, electrostatic bridging with mucosal proteins, and pH-triggered contraction that stabilizes adhesion under strong acidity. It is worth noting that the appropriate hemostatic material should be selected based on the bleeding pathology.

#### 3.2.2 Gastric perforation

Gastric perforation is one of the most serious diseases of the digestive system, which refers to the complete damage to all four layers of the stomach wall and penetration into the abdominal cavity 74, 75. After gastric perforation, the entry of gastric contents into the abdominal cavity can lead to gastric fluid leakage into the cavum abdominis, abdominal adhesions, bacterial peritonitis, and even death 3, 76. Surgical suturing is a common method to treat gastric perforation, but it can cause secondary damage to the gastric wall 77. Therefore, it is crucial to design an adhesive material that avoids suturing for repairing gastric perforation. Research has shown that the mortality rate of rabbits with gastric perforation treated with sutures and cyanoacrylate was 66%, while all rabbits treated with the light-controlled adhesive patch survived 78. To secure normal gastric peristalsis and avert contraction at the anastomotic site, the optimal adhesive for treating gastric perforation should exhibit strong elasticity, adhesiveness, fatigue resistance, and flexibility 79.

**Figure 5:**
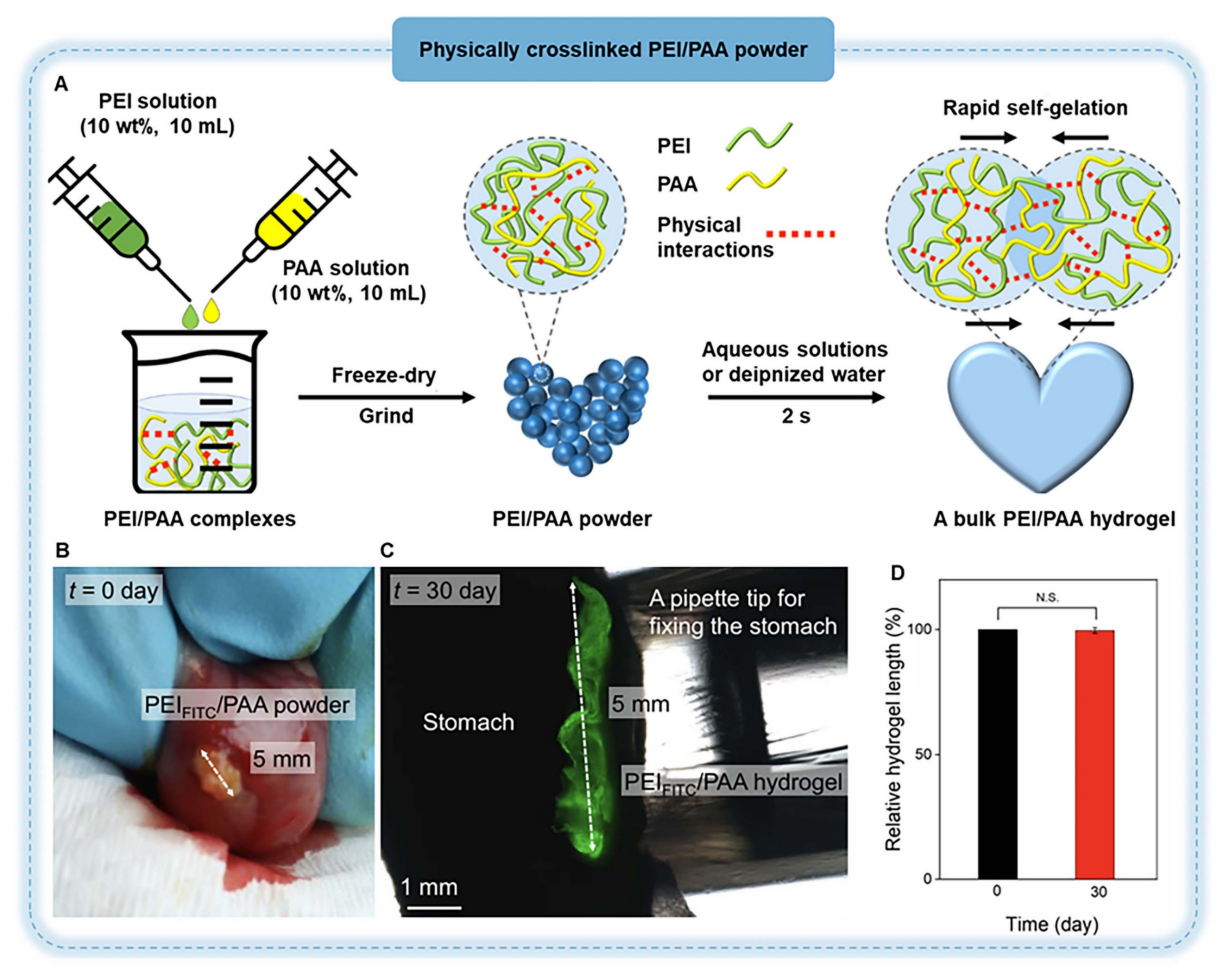
Hydrogel powder for gastric perforation. (A) Schematic illustration of PEI/PAA powder and PEI/PAA hydrogel for gastric perforation. (B) Photos of a PEI_FITC_/PAA hydrogel on the serosa of the stomach on day 0 and (C) day 30 after applying PEI_FITC_/PAA powder. (D) *t*-test suggesting the hydrogel remains stable during the tested period. FITC: fluorescein isothiocyanate used in hydrogel staining. Adapted with permission from 81, copyright 2021, American Association for the Advancement of Science.

**Figure 6:**
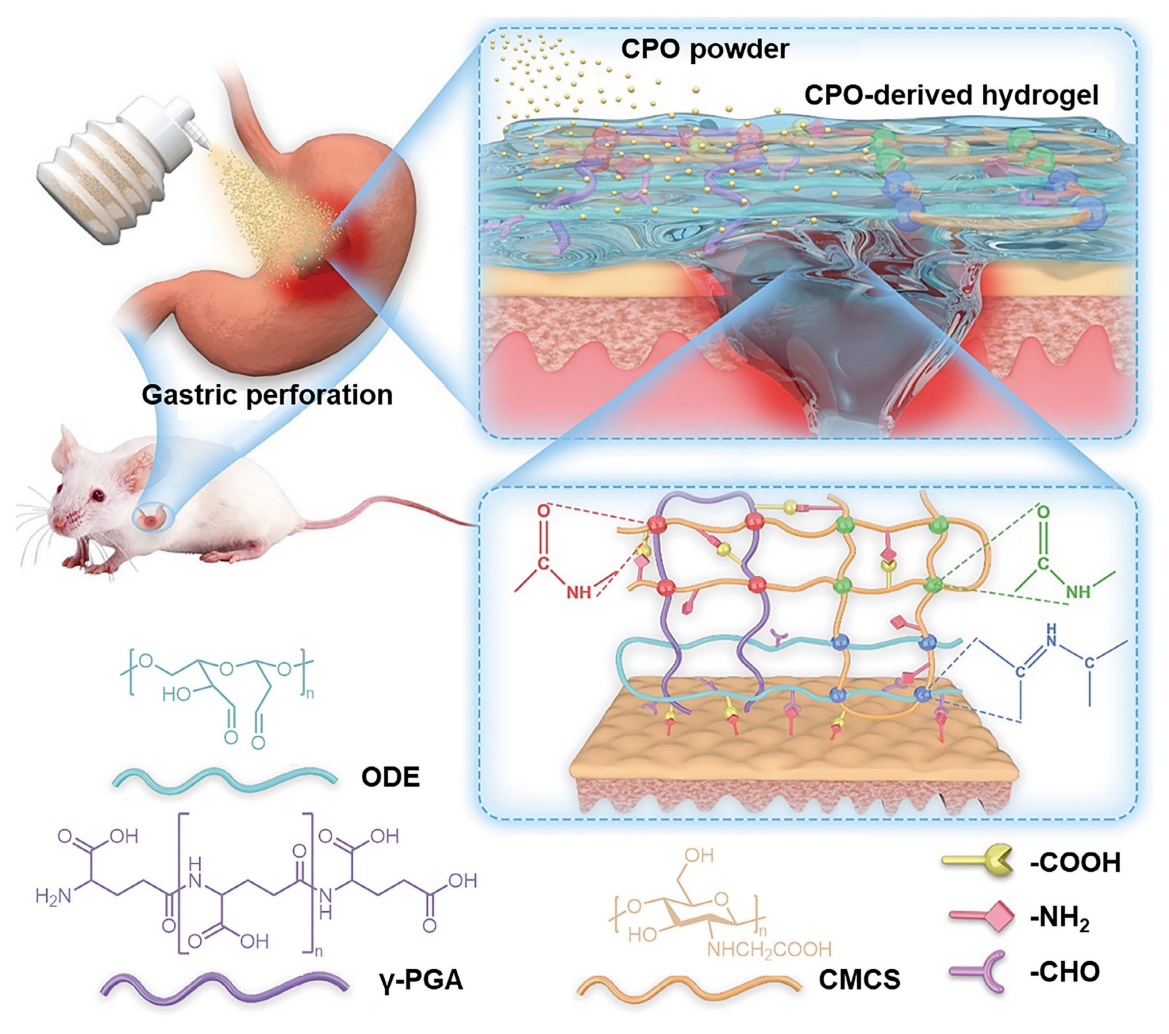
Schematic diagram of CPO hemostatic powder for the gastric perforation model in rats and its chemical structure. Adapted with permission from 82, copyright 2024, Elsevier.

Traditional hydrogel adhesives are typically in a gel state with a wet surface, and wet hydrogels are inconvenient for storage and transportation. If hydrogel adhesives are made into a powder, this problem can be resolved. A hybrid dry powder (HDP) strategy is reported that features rapid gelation of the powder upon contact with water and achieves tissue adhesion, enabling rapid sealing of wet tissue 80. Peng *et al.* 81 reported a hydrogel powder consisting of PEI and PAA, which can rapidly gelatinize *in situ* by adsorbing interfacial water within 2 seconds via physical interactions between the polymers (Figure 5A). Owing to the slow healing of gastric perforation, secondary perforation occurs very easily 74, and the retention time of hydrogel adhesives at the site of gastric perforation is particularly important. The PEI/PAA hydrogel remained stable for 30 days after implantation in the stomach, with no noticeable degradation, which indicates that it can provide long-term protection for the perforated part of the stomach until the complete healing of the perforated part (Figure 5B-D). Another type of hemostatic powder, such as carboxymethyl chitosan/poly γ-glutamic acid/oxidized dextran (CPO) powder, can also be used to block gastric perforation (Figure 6) 82. When gastric perforation occurs, CPO powder is sprayed onto the affected area. This powder can absorb interstitial fluid from the blood near the wound, thereby concentrating coagulation factors and platelets and achieving rapid hemostasis. Meanwhile, Schiff base and amide bonds were formed, and the hydrogel was gelatinized within 15 seconds. In addition, the abundant -NH_2_ groups in the tissue react with the -CHO group of ODE via a Schiff base reaction to form a strong bond, creating a physical seal that prevents bleeding.

**Figure 7:**
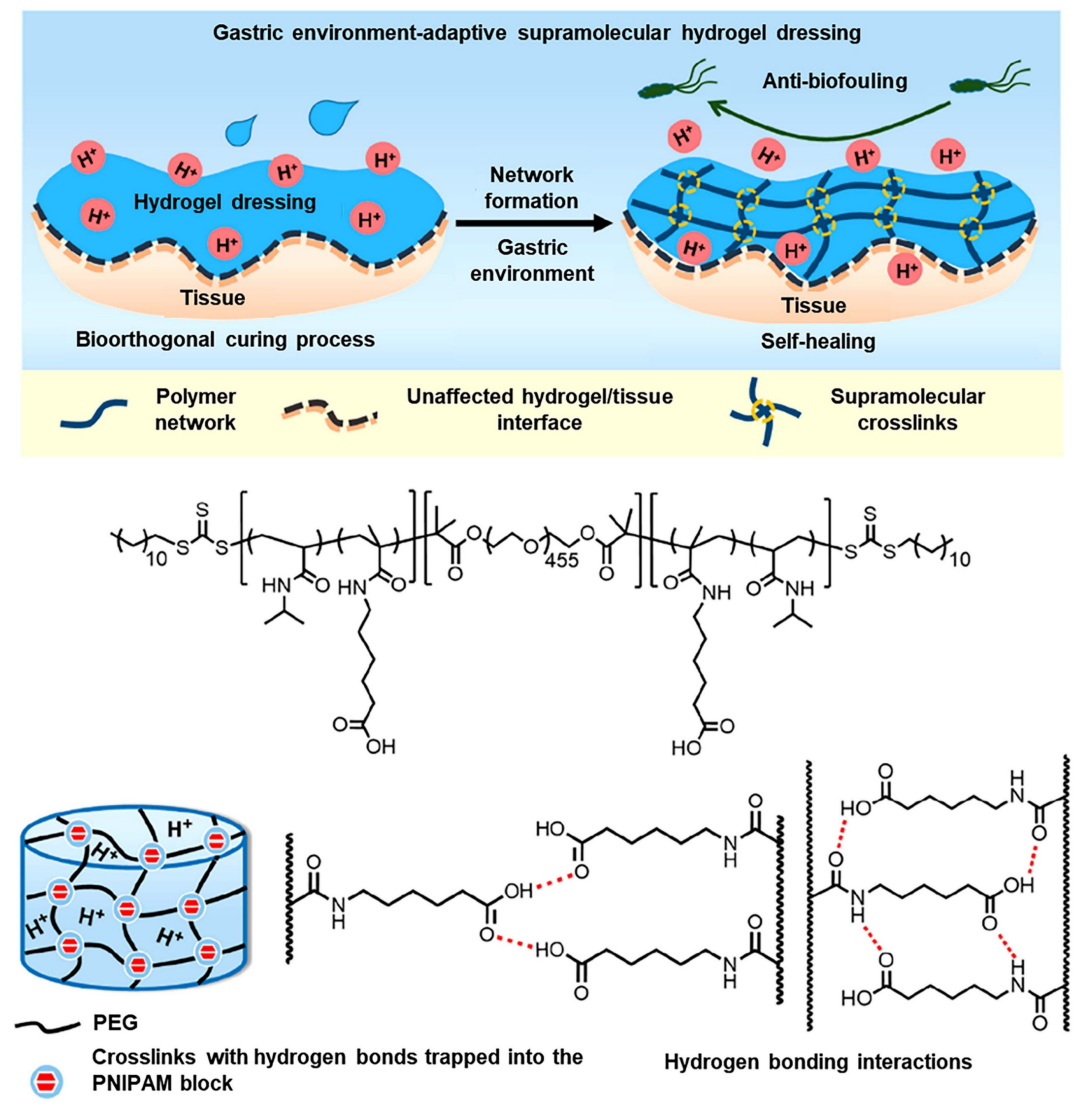
Schematic diagram of the gastric environment-adaptive supramolecular hydrogel dressing for gastric perforation and chemical structure of the hydrogel and supramolecular hydrogel network structure. Adapted with permission from 83, copyright 2021, American Chemical Society.

Owing to the special anatomical position of the GI tract, the hydrogel used in the GI tract must be delivered to the wound site via endoscopy, so injectability is essential. Wang *et al.* 83 proposed an injectable hydrogel-related supramolecular assembly of ABA triblock copolymer for repairing gastric perforation in rats (Figure 7). The ABA triblock copolymer consists of an intermediate hydrophilic PEG block and a terminal temperature-reactive poly(N-isopropylacrylamide) (PNI-PAM) block, with pH-sensitive acryloyl-6-aminocaproic acid randomly incorporated 83. The hydrogel dressing is liquid at 4 ℃ and forms a hydrogel at physiological temperature, allowing it to be readily injected into the required position.

Due to hydrophobic interactions and hydrogen bonds between the materials, this hydrogel has the ability to rapidly self-heal, allowing it to repeatedly repair damage to the hydrogel dressing caused by gastric peristalsis 83. Besides, the supramolecular hydrogel dressing can prevent biological contamination, thereby reducing the incidence of infection after gastric perforation and accelerating its repair 83. Compared with a single syringe, a double syringe can separate the polymer from the crosslinking agent and control *in situ* gelation of the hydrogel at the desired location. Chen *et al.* 84 proposed an injectable dual-network hydrogel formed *in situ* using a double syringe to separate the polymer and crosslinking agent. The hydrogel can take shape after extrusion from the micro mixer, which is connected to the double syringes. It has strong adhesion, toughness, adaptability, degradability, and biological activity, which can effectively seal GP and promote the regeneration of gastric mucosa. Other injectable hydrogels are also reported, such as a hydrogel based on cation-π interactions between protonated amines and aromatic rings 85, hydrogel adhesive AN@CD-PEG&TQ with therapeutic and biological imaging functions 86 and so on.

**Figure 8:**
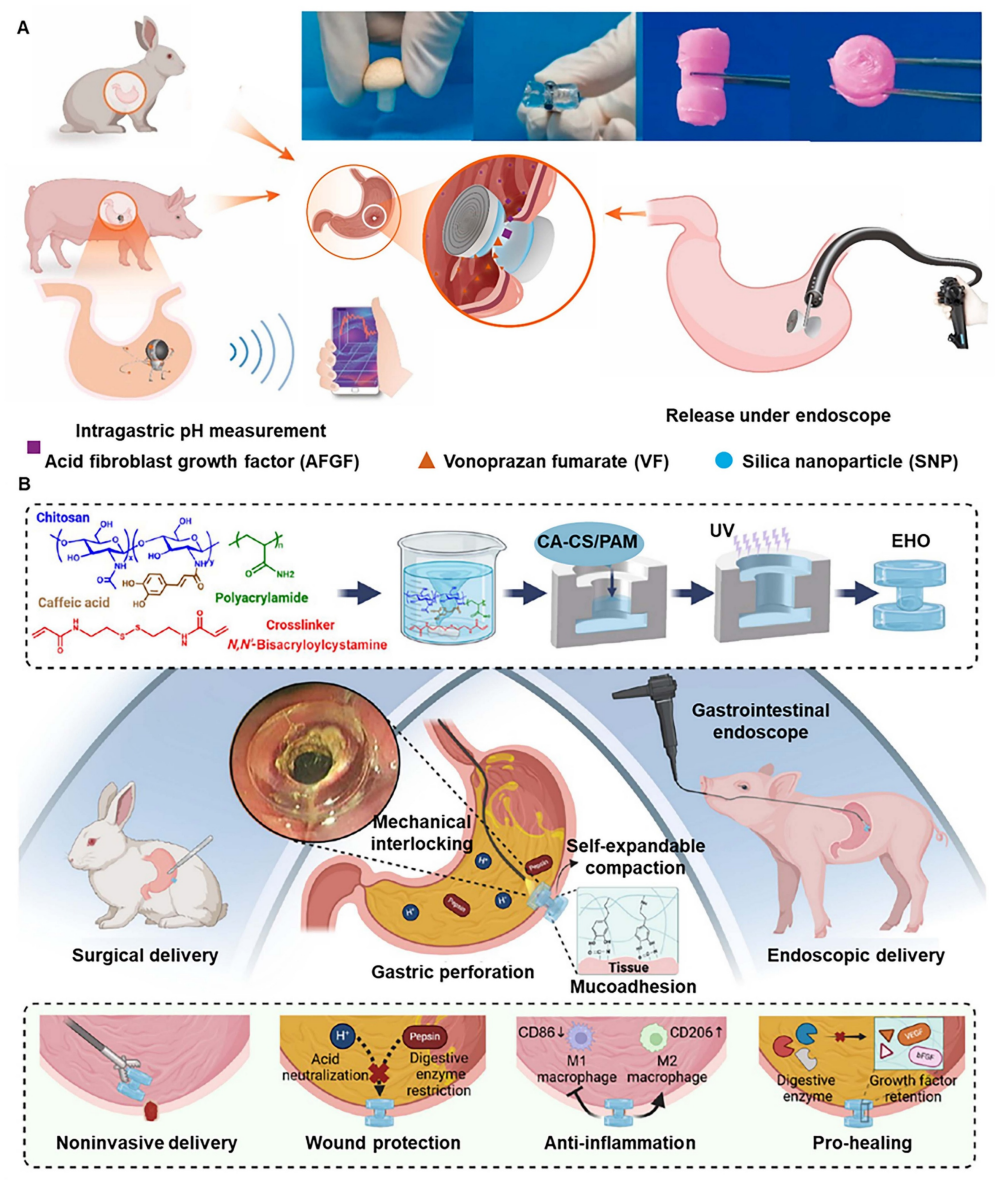
Hyperboloid hydrogel occluders for gastric perforation. (A) Schematic diagram of a compressible hyperboloid hydrogel inspired by a compressible mushroom-cap-inspired device for the treatment of gastric perforation. Adapted with permission from 88, copyright 2022, American Chemical Society. (B) Schematic diagram of the preparation of EHO and its application in endoscopic repair of gastric perforation. Adapted with permission from 89, copyright 2025, American Chemical Society.

Patients with severe gastric perforations often require surgical suturing for repair, which can cause secondary tissue damage. Xing *et al.* 87 designed a sutureless hydrogel for gastric surgery, which is composed of gelatin methylate (GelMA) and tannic acid (TA). TA provides abundant hydrogen bonds in this hydrogel, resulting in excellent adhesion performance, ultimate stress, compression modulus, and elongation. Inspired by the shape of a mushroom cap-stick, Liu *et al.* 88 designed a medium-CBA cross-linked hydrogel (MCH) with silica nanoparticles (SNPs) coating, which remained at the defect for 4 weeks and enhanced mucosal and vascular generation via loading acidic fibroblast growth factor (AFGF). Moreover, a built-in wireless pH capsule maintained intragastric pH above 4 for >83% of the time, minimizing rebleeding risk (Figure 8A). Similarly, inspired by cardiac occluders, Linghu *et al.* prepared a double umbrella hydrogel occluder (EHO) composed of caffeic acid (CA) grafted CS and polyacrylamide, which can be used for endoscopic delivery and effectively seal gastric perforation through hydrogen bonding and chelation between tissue and polymer, and expansion of hydrogel (Figure 8B) 89. Under the action of gastric acid, the catechol groups in CS and caffeic acid undergo nucleophilic reactions, endowing EHO with the ability to resist gastric acid and enzyme invasion, thereby promoting gastric perforation healing. At the same time, the EHO group had higher M2 macrophages and lower M1 macrophages at the site of gastric perforation, and the expression of bFGFp and pro-angiogenic VEGF was enhanced. This material has excellent anti-inflammatory effects.

**Figure 9:**
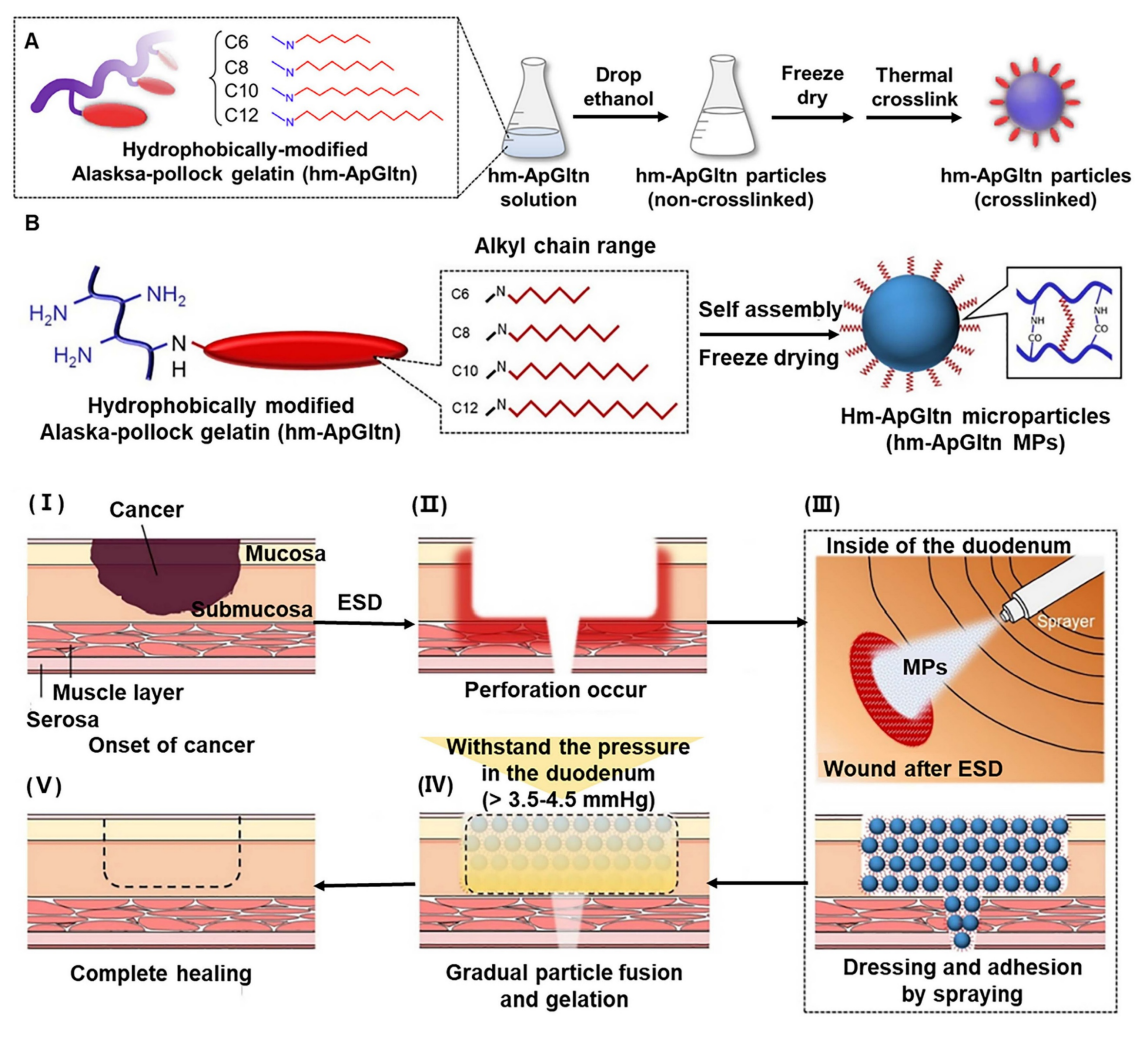
Hydrogel with hydrophobic properties. (A) Structure and preparation process of ApGltn hydrogel. Adapted with permission from 91, copyright 2019, Elsevier. (B) A coacervation method for the formation of hm-ApGltn modified with various alkyl chains, and the preparation of hMPs by freeze drying and thermal crosslinking. (I) Cancer growing onto the mucosa and submucosa. (II) Perforation due to inflammation in post-ESD. (III) hMPs are sprayed on the wound tissue. (IV) hMPs swell and form the hydrogel to close the perforation. (V) hMP hydrogels degradation and wound healing. Adapted with permission from 32, copyright 2021, Elsevier.

The introduction of hydrophobic substances into hydrogel adhesives can effectively attenuate the hydration barrier between hydrogels and hydrated tissue, thereby ensuring a relatively tight connection between them 33. Taguchi *et al.* 90 increased the hydrophobicity of the hydrogel by modifying the long alkyl chain and developed a hydrogel adhesion consisting of dodecyl-modified Alaska pollock gelatin (ApGltn) and 4S-PEG. The amino residue of ApGltn is modified by a dodecyl group to form a hydrophobic group, which enhances its sealing effect on wet tissues 90, 91. Animal experiments have shown that a C12-ApGltn-based hydrogel injected into the subcutaneous tissue of rats degrades within 28 days without eliciting an inflammatory response 90. Consequently, C12-ApGltn-based sealant has excellent potential for application in various surgeries. Due to its underwater adhesion stability, the research group also used hydrophobically-modified ApGltn to treat GI tract wounds after ESD (Figure 9A) 91. Contact angle measurements indicate that the surface hydrophobicity of particles modified with long alkyl chains is significantly increased, with C10-MPs having the highest hydrophobicity. Based on hydrophobically modified ApGltn, the research team made spray-hydrophobized microparticles (hMPs) as a wound dressing via coagulation, freeze-drying, and thermal crosslinking, which exhibit high tissue adhesion under wet conditions (Figure 9B) 32. The sprayable tissue adhesive hMPs can close perforations after ESD or prevent delayed perforation after ESD. However, hydrophobic designs alone do not yield optimal wet adhesion because, even with hydrophobic pairs, contact remains incomplete due to small puddles formed by rough nanoscale channels 92. Therefore, rapid dehydration and subsequent removal of residual water are critical to achieve strong noncovalent adhesion to the wet surface. A half-dry adhesive was reported that used PAA to form a covalent cross-linking network and silk fibroin (SF) to create a secondary semi-interpenetrating network 33. When encountering wet tissue, the adhesion can quickly repel liquids and then adsorb residues under external pressure, thereby eliminating the influence of liquids on adhesion. Due to the excellent adhesion to wet tissue, hydrophobically modified hydrogels can achieve local sustained release of anti-cancer drugs when loaded with them, thereby removing residual cancer tissue after endoscopic surgery 93.

**Figure 10:**
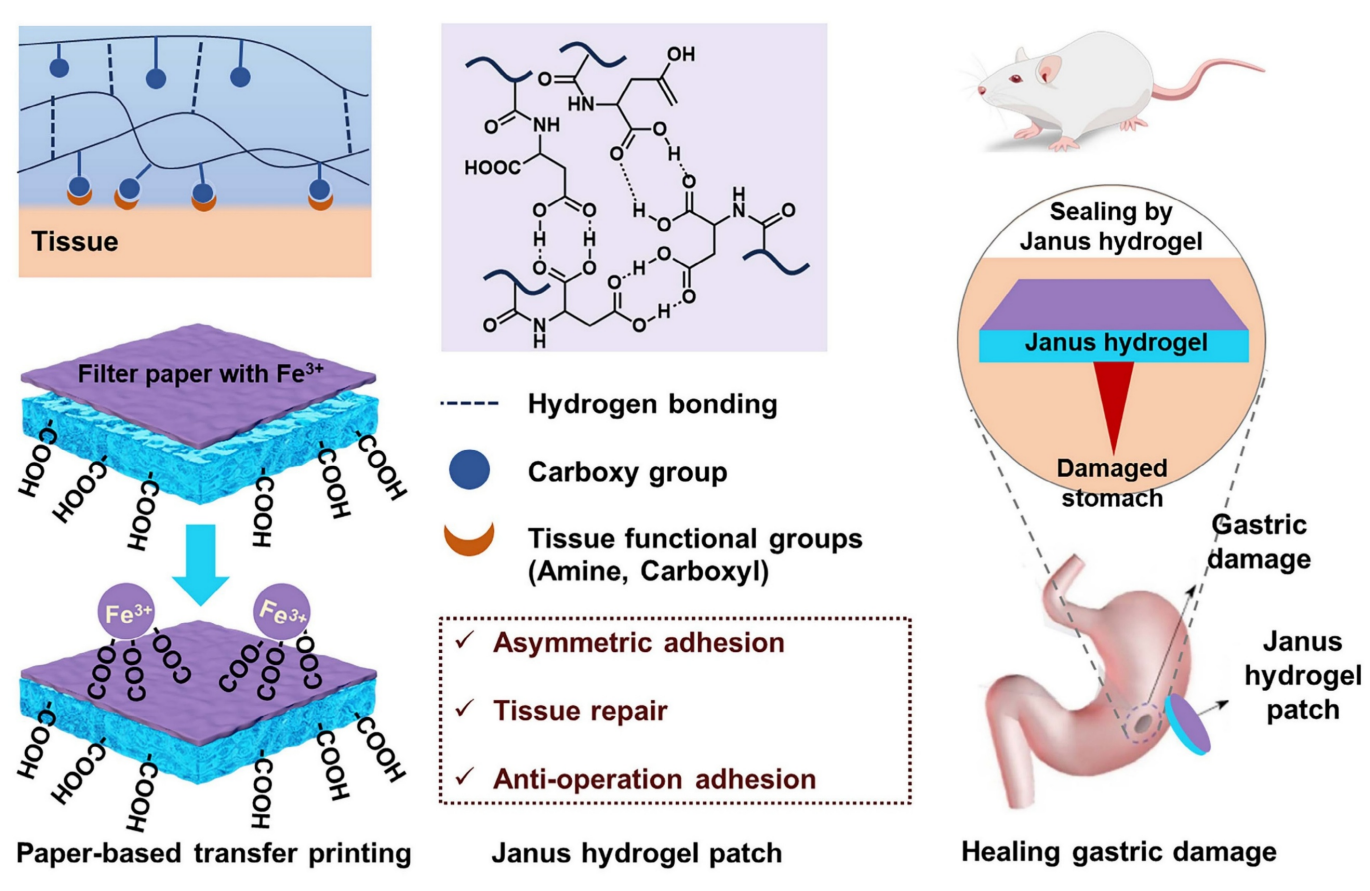
Schematic diagram of PAASP hydrogel adhesion to tissue via hydrogen bond crosslinked network and Janus hydrogel patch prepared by paper-based Fe^3+^ transfer printing for gastric perforation repair 77.

**Figure 11:**
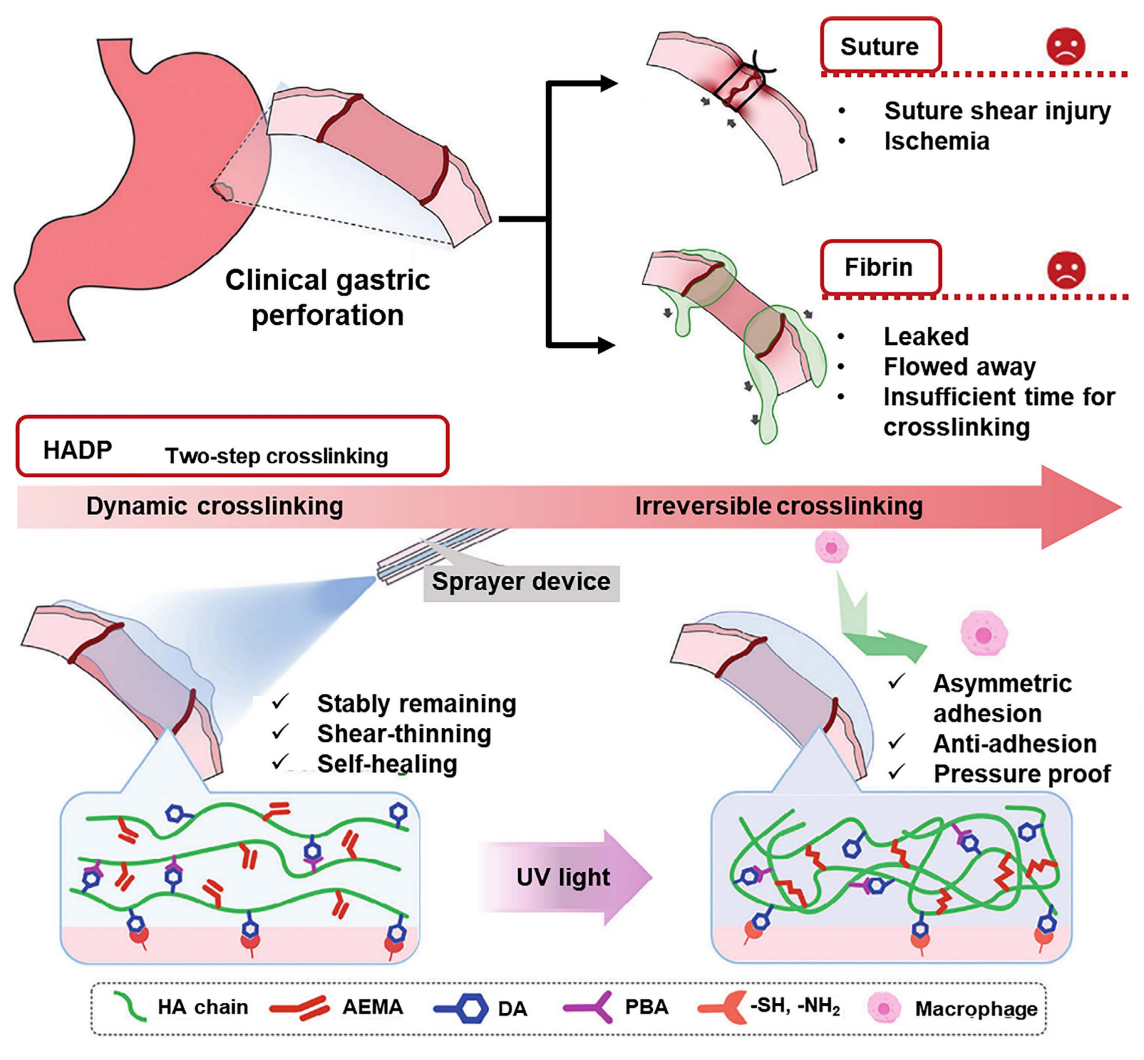
Schematic diagram showing the disadvantages of surgical sutures and fibrin as materials for closing gastric perforations and the realization of asymmetric adhesion of HADP hydrogel via the dynamic reversible boronate ester bonds and irreversible crosslinking after UV light. Adapted with permission from 31, copyright 2024, Wiley-VCH GmbH.

The above conventional double-sided biological adhesives have a significant clinical limitation: their uncontrolled adhesion can lead to postoperative adhesions, severely limiting their clinical application in wound repair, especially in abdominal surgery. The formation of postoperative adhesions will lead to serious consequences, including chronic pelvic inflammation, intestinal obstruction, and infertility, typically requiring readmission or surgery 94, 95. Accordingly, designing and synthesizing a single-sided adhesive is particularly important. The Janus hydrogel patch is a single-sided adhesive hydrogel that can prevent postoperative adhesion. One side has strong adhesion, which can be used to paste the injured tissue to promote healing, and the other side has no adhesion to prevent postoperative adhesions 77, 96. The carboxyl groups of polymeric N-acryloyl aspartic acid (PAASP) not only form hydrogen bonds with polar groups at the tissue surface, demonstrating strong adhesion to injured tissues, but also bind with metal ions to form non-adhesive interfaces, avoiding peritoneal adhesion during tissue repair *in vivo* (Figure 10) 77. The authors achieved the function through paper-based Fe^3+^ transfer printing technology. The experiment showed that the Janus hydrogel patch can simultaneously achieve tissue repair and prevent postoperative adhesion. Moreover, the negatively charged carboxyl groups in the hydrogel can be electrostatically bound to the cationic oligosaccharide via unilateral impregnation to form a new Janus hydrogel, whose two sides show significantly different adhesiveness 96. However, such hydrogels are challenging to use in minimally invasive surgeries because they cannot be administered through injection. In contrast, Wu *et al.* proposed a photocurable injectable hydrogel (HAD) that grafts catechol and methacrylate onto hyaluronic acid, using LAP as a photo-initiator. The HAD precursor solution contains plentiful catechol groups, which can form hydrogen bonds with protein residues of the injured tissue, thereby allowing the precursor solution to adhere to the tissue surface 97. Under UV curing, the external surface of HAD hydrogel shows antiadhesion due to the formation of 2-aminoethyl methacrylate hydrochloride (AEMA) network that restricts the free circulation of catechol groups, so as to prohibit the interaction between the catechol groups on the HAD hydrogel and the amino groups on the tissue. In addition, the precursor solution of HAD hydrogel exhibits good shear-thinning properties, enabling endoscopic injection and adaptation to complex, irregular tissue surfaces. However, due to the low viscosity of HAD precursors, they cannot remain stably on the wound surface. Therefore, the group added hyaluronic acid grafting phenylboronic acid (PBA) groups to the HAD hydrogel, which ensures the adherence of the hydrogel to irregular or inclined surfaces upon delivery while preserving its injectability (Figure 11) 31. There are other types of Janus hydrogels, e.g., Liang *et al.* 98 designed an asymmetric adhesion hydrogel induced by microparticle deposition composed of acrylic acid, chitosan, tannin, and Al^3+^, whose asymmetric adhesion is effectuated by the deposition of calcium alginate microspheres on the bottom of the hydrogel. Chen *et al.* 99 constructed a double-sided tissue-adhesive hydrogel, which integrates onto the bottom surface of the acellular dermal matrix. Li *et al.* 100 developed an adhesion-triggered hydrogel system consisting of polyacrylamide-alginate (PAM/SA) hydrogel and tannic acid (TA). TA contains rich polyphenol structures and acts as an adhesion-trigger molecule, which can form plentiful hydrogen bonds at the tissue interface, achieving rapid and strong adhesion to the surface of wound tissue. Besides, Liu *et al.* prepared the Tempo FPF hydrogel based on dynamic iminoborate and borate bonds, which can not only seal gastric perforation, but also prevent organ adhesion 101.

When gastric acid is high, the hydrogel swells readily, leading to a mismatched adhesion between the hydrogel adhesives and the gastric mucosa, further impacting the adhesive effect 102. To solve the issue, Yuan *et al.* 103 proposed a double-network hydrogel strategy based on ionic nanoreservoirs (INR) that can persist prolonged adhesion. The first polyacrylamide network cross-links on gastric tissue within 5 seconds after exposure to blue light and improves adhesive properties through mechanical interlocking 103. As an INR, nanohydroxyapatite can gradually release Ca^2+^ in an acidic environment and form a second network with SA to restrict the expansion of hydrogels in gastric juice 103. In addition, an acid-resistant hydrogel (ATGel) adhesive was developed, achieving immediate, effective sealing of a gastric perforation within 2 seconds 13. Compared with hydrogel adhesives used for hemostasis of gastric ulcers, the hydrogel used for perforation repair relies more on mechanical strengthening mechanisms. For instance, postoperative tissue adhesion is prevented through strategies such as dual-network structures, hydrophobic interlocking, and asymmetric adhesion.

#### 3.2.3 Endoscopic submucosal dissection (ESD)

Endoscopic resection includes endoscopic mucosal resection (EMR), ESD, endoscopic submucosal tunnel resection, and trap-assisted endoscopic resection 104, 105. With the evolution of endoscopic technology, an increasing number of GI diseases can be treated through endoscopy, including early gastric cancer, gastric polyps, and intragastric masses 106-109. Nevertheless, endoscopic surgery also has some disadvantages, such as incomplete resection, perforation, and the risk of bleeding 110, 111. The key method to reduce the risk of endoscopic resection is to inject a solution under the mucosa to form a submucosal fluid cushion 112. Submucosal fluid cushions can elevate lesions, creating sufficient operating space between the mucosa and intrinsic muscle layer, thereby reducing iatrogenic risks 113. Physiological saline is one of the most ordinary fluids used as a submucosal injection, but it is easily absorbed by surrounding tissues, resulting in a submucosal fluid cushion that can only last for a short period of time 114, 115. The best submucosal injection material should have injectable properties and form a thick, durable submucosal cushion after injection, supporting the diseased mucosa and adhering to the wound to accelerate wound healing after the diseased mucosa is removed. Hydrogels are suitable as submucosal injection materials because of their excellent biocompatibility, appropriate hardness, and low diffusivity. To achieve low viscosity and injectability during injection, while forming a solid gel after injection, some hydrogels with special properties are designed, including temperature-sensitive hydrogels, photo-crosslinked hydrogels, hydrogels with a 2-step injection system, and shear-thinning hydrogels.

By mixing sodium glycerophosphate (GP) with the CS solution, a CS/GP hydrogel was developed 116. It is a viscous liquid at lower temperatures, such as room temperature or below, and can be converted into solid hydrogels at physiological temperatures near 37 ºC, which means that it has clear injectability at an appropriate temperature. The outcomes of animal experiments indicated that the CS/GP system was an effective submucosal injection, and the required amount in ESD surgery was much less than that of physiological saline 117. Shan *et al.* 118 introduced collagen into the CS/GP hydrogel to construct the chitosan/β-glyceryl phosphate/collagen (CS/GP/Col) system, which rapidly gelled at 37 ºC and kept flowing for a long time at low temperature, and could effectively stop bleeding. Hydroxypropyl cellulose (HPC) has excellent biological adhesion properties 119. Tang *et al.* added HPC to the CS/GP/Col hydrogel to strengthen the adhesion and biocompatibility of the hydrogel, which made the hydrogel system more suitable for ESD 120. Nevertheless, the application of CS/GP/Col hydrogel for submucosal injection is challenging because of its reduced fluidity at low temperatures 121. To address this disadvantage, Ni *et al.* 121 utilized lactic acid (LA) to modify chitosan, thereby enhancing the fluidity of CS/GP/Col hydrogel at low temperatures and creating a new hydrogel system. Liu *et al.* 122 made the hydrogel into a lyophilized powder. Compared with the hydrogel, the powder has better low-temperature fluidity and significantly increased injectability 122, 123. The concentration of the chitosan solution affects the injectability of the hydrogel precursor solution and gelation time, making it difficult to achieve a balance. Liu *et al.* 124 prepared a chitosan solution with a high pH value (HpHCS; pH 6.19), which can maintain rapid gelation at a low concentration and improve injectability. However, as the concentration of HpHCS decreased, the precursor solution of the hydrogel became more and more turbid, and the hydrogel became more and more brittle. Subsequently, the team added polyvinylpyrrolidone (PVP) to the system to form a strong hydrogen bond with CS, making the hydrogel more elastic 125. Experiments showed that HpHCS-5%-PVP-GP hydrogel effectively improved the height of the submucous fluid pad 125. In addition, other types of temperature-sensitive hydrogels are also used for ESD, such as a FS hydrogel based on a mixture of polyethylene-polypropylene glycol (F-127) and SA 126 and a hydrogen-bond-driven conductive thermosensitive hydrogel based on polystyrene sulfonate and F-127 127.

In addition to thermo-sensitive hydrogels, photocrosslinked hydrogels can also be used as submucosal liquid cushions for ESD. Chitosan hydrogel containing azide and lactose (Az-CH-LA) can be coagulated by ultraviolet irradiation, injected into the submucosa before ESD, and then irradiated with ultraviolet light, producing significant mucosal bulges and significantly reducing bleeding after mucosal resection 128. Compared with hypertonic saline, photocrosslinked chitosan hydrogel (PTH) can make more durable submucosal bulges with clearer edges, which are more conducive to the accurate development of ESD. According to histological analysis, PCH can undergo complete biodegradation within 8 weeks without adverse effects on biological systems 129.

The two-step injection method is also effective for obtaining a high-mucosal protrusion liquid pad. In a two-step injection system, two low-viscosity solutions are injected separately into the submucosa, where they crosslink to produce a high-viscosity submucosal pad 130. Fan *et al.* reported a naturally derived gelatin-oxidized alginate saline gel (G-OALG), which used an endoscopic double-needle/two-step injection needle to inject gelatin and alginate oxide into the esophageal submucosa, and via the formation of Schiff base, a good submucosal liquid pad was obtained 113.

**Figure 12:**
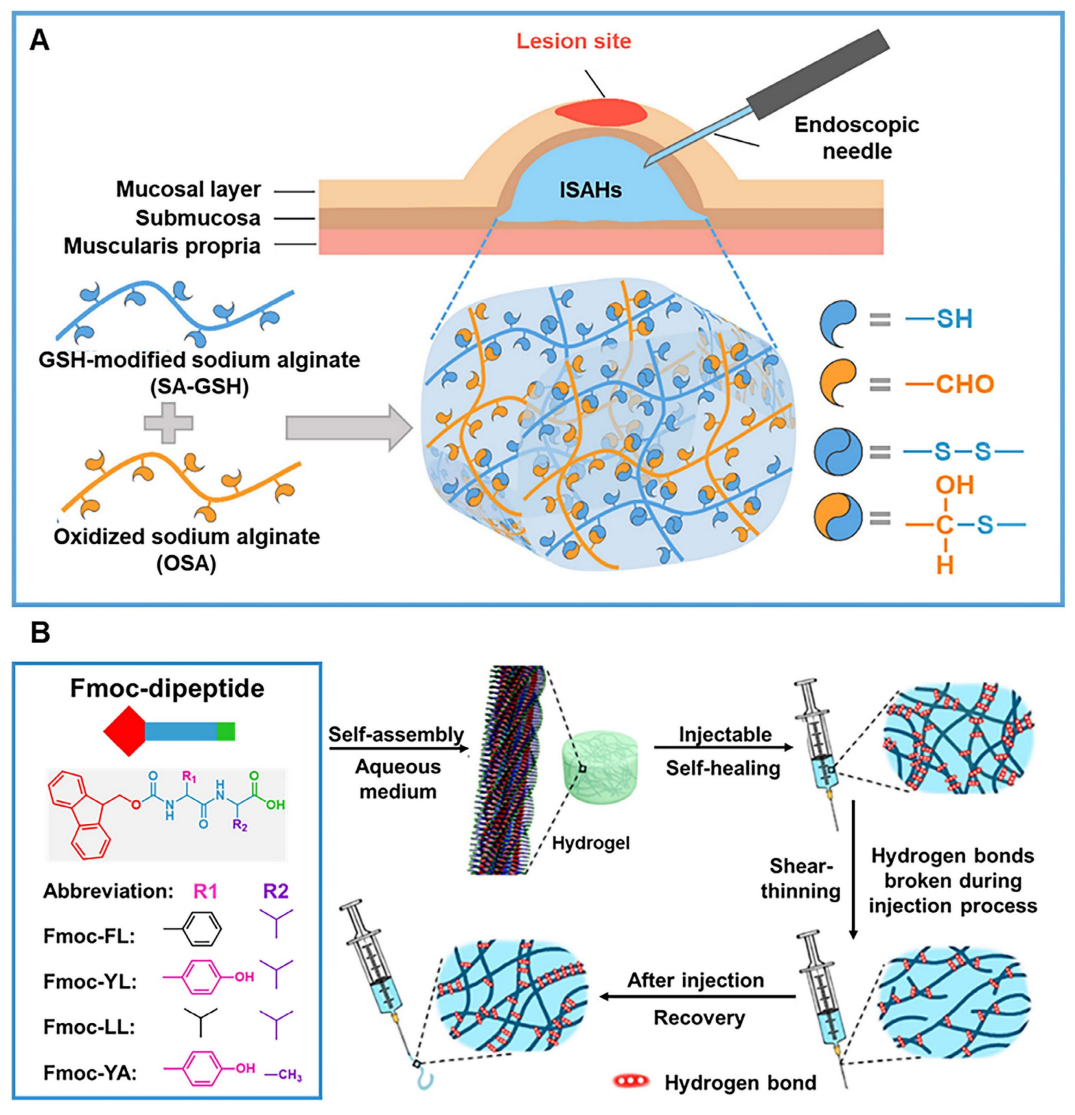
Shear-thinning hydrogels for ESD. (A) Schematic diagram displaying the synthesis mechanism and application of the injectable sodium alginate hydrogels (ISAHs). Adapted with permission from 132, copyright 2022, Elsevier. (B) Schematic illustration displaying Fmoc-dipeptide self-assembly hydrogels with shear-thinning and self-healing properties. Adapted with permission from 133, copyright 2020, American Chemical Society.

Temperature-sensitive hydrogels can clog endoscopic needles, and photocrosslinkable hydrogel curing requires an ultraviolet light source, which is challenging to use within an endoscope. Furthermore, the two-step injection method is complicated, making it an unnecessary burden. Therefore, shear-thinning hydrogels began to be used as submucosal fluid cushions. When shear force is applied to the shear-thinning hydrogel, it can rapidly transform into a low-viscosity sol, reducing the injection pressure. After injection, the shear-thinning hydrogel can reform a cross-linking network, and completely restore the solid hydrogel morphology 131. Gellan glue hydrogel (GGH) formed through double-spiral aggregation at a cross-linking point exhibits shear-thinning hydrogel properties 131. Its injection pressure is equivalent to the injection pressure of a small molecule solution when injected through the endoscopic injection needle. Ma *et al.* 132 prepared an injectable shear-thinning hydrogel for ESD, composed of thiolated sodium alginate (SA-GSH) and OSA (Figure 12A). The shear-thinning property enables this hydrogel to be injected at low pressure. When this hydrogel is injected into the submucosa, the cross-linked network composed of disulfide bonds and thioacetals again forms, and the submucosa elevation generated by the hydrogel is 13% -18% higher than that of commercial ESD injection solution 132. Ren *et al.* 133 used a range of aromatic dipeptides, such as 9-fluorenylmethoxycarbonyl (Fmoc)-conjugated phenylalanine-leucine (FL), tyrosine-leucine (YL), leucine-leucine (LL), and tyrosine-alanine (YA), to prepare self-healing and shear-thinning hydrogels, explored their self-assembly behaviors and regulated the mechanical strength (Figure 12B). Compared to commercial ESD fillers such as physiological saline and commercial polymer PF-127, Fmoc YL hydrogel is more suitable for ESD as a filler 133. In addition, hydrogel composed of the anionic sodium carboxymethyl starch (CMS) and cationic LAP prepared by Wang *et al.* 134, the CS hydrogel with Ag nanoparticles 135, synergistic drug-loaded shear-thinning hydrogel 136, thermogel generated by the “block blend” strategy 137, alginate/Laponite hydrogel 138, the polyglycerol stearate hydrogel (PGSH) 139 and the hydrogel based on maleimide-based OSA and sulfhydryl carboxymethyl-chitosan 140, all can effectuate persistent improvement of the mucosal cushion by endoscopic injection. Additionally, Liu *et al.* 141 prepared succinylated hydroxybutyl chitosan (HBC) hydrogel with excellent temperature sensitivity, *in situ* gelation at 37 °C, and can also be injected through the endoscope injection needle after hydrogel formation, effectively avoiding the risk of clogging the needle tube. Therefore, unlike hydrogel adhesives used for gastric ulcer and perforation therapy, submucosal injection hydrogels primarily form stable mucosal pads via mechanisms such as shear-thinning behavior, temperature- or light-triggered sol-gel transitions, and controllable osmotic swelling. These hydrogels usually exhibit excellent injectability and shape retention rather than strong interfacial adhesion.

In conclusion, for gastric bleeding, rapid gelation and acid-resistant hydrogels can quickly adhere to bleeding sites and provide strong interfacial stability. For gastric perforation repair, hydrogel adhesives are needed to provide sufficient mechanical strength to withstand gastric peristalsis and maintain long-lasting sealing. In ESD surgery, hydrogels are typically used as submucosal cushions, requiring excellent injectability and shape retention. Postoperative esophageal stenosis is often accompanied by inflammation. Therefore, hydrogel adhesives loaded with cells or exosomes can be used to regulate inflammatory responses and inhibit fibrosis. Notably, due to individual differences among patients and the dynamic fluctuations in the microenvironment, real-time monitoring and timely adjustment of hydrogel adhesive selection are often required in clinical treatment.

### 3.3 Pancreatic cancer

Pancreatic cancer is the sixth most common cause of cancer-related deaths worldwide. It is estimated that 510,566 patients were newly diagnosed with pancreatic cancer and 467,005 patients died of pancreatic carcinoma in 2022 worldwide 34. Because of the absence of typical clinical symptoms in patients with early pancreatic cancer, the majority of patients were already in the advanced stage when diagnosed, and the five-year relative survival rate was only 10% 142. Currently, radiotherapy is considered the standard therapy for patients with pancreatic cancer, particularly those with localized advanced cancer who have responded to chemotherapy for the first six months, are in a stable condition, have experienced unacceptable chemotherapy-related toxicity or have deteriorated due to chemotherapy toxicity 143. Recent research has shown that increasing the dose of radiotherapy can improve overall survival in patients 144. However, the main problem with an increased radiation dose is the toxicity to contiguous organs at risk (OARs), especially the duodenum, which is close to the pancreas 145. Ding *et al.* 146, 147 used an absorbable radiopaque hydrogel spacer for spacing the OARs from the head of the pancreas through endoscopic ultrasound guidance in a cadaveric model. Subsequently, the group demonstrated the feasibility and safety of injecting the hydrogel spacer in the porcine model, human trial and multi-site feasibility cohort study 147-149 and proposed a dose prediction model to identify patients who require placement of a hydrogel spacer before radiotherapy to meet predefined dose constraints 150. The team developed a duodenal spacer simulator platform and systematically studied the dosimetric effects of spacer positions 145.

## 4. Challenges and perspectives

Although hydrogel adhesives show great promise for GI wound healing, several challenges remain, including durability in acidic environments, mechanical robustness under peristalsis, and long-term safety. Additionally, delivery issues such as blocking of injection needles or spray nozzles during application still pose practical difficulties. The integration of hydrogel materials with nanomaterials and controlled drug-release systems offers promising opportunities to address the above issues. Overcoming these obstacles will be crucial for the clinical translation of hydrogel adhesives in digestive tract wound healing.

For hydrogel adhesives used for gastric bleeding and ulcers, the ingredients that play a therapeutic role are relatively simple and cannot address serious complications. Hydrogels used in submucosal injection materials usually have good physical properties that can provide an ideal height of mucosal bulge, but their therapeutic functions have been largely overlooked. Key issues, such as accelerating postoperative wound healing and preventing postoperative infections, have not been addressed. The introduction of nanomaterials, small-molecule drugs, peptides, and proteins to optimize the therapeutic function of the hydrogel adhesive is one of the most direct means; they have a significant therapeutic effect on gastric wound healing and its complications. Therefore, a reasonable combination of the above therapeutic ingredients and hydrogels to achieve controlled drug release and functional synergy between drugs and hydrogels is a significant future development direction for hydrogel adhesives. Lin *et al.* 151 uniformly coat covalent organic frameworks (COFs) on oxidized carbon nanotubes (OCNTs) and coordinate with Fe^3+^ to obtain nanocomposites O@CF. O@CF can participate in a Schiff base reaction via its surface residues to crosslink with oxidized dextran and with chitosan grafted with caffeic acid, yielding a hybrid hydrogel (O@CF@G). The hybrid hydrogel can improve the mechanical properties of the original hydrogel and exhibit multifunctionality through O@CF, including peroxidase (POD) and catalase (CAT) activities and photothermal properties. Therefore, it can effectively eliminate drug-resistant biofilms, kill bacteria, alleviate hypoxia in diabetic wounds, enhance transmission of intercellular electrical signals, and ultimately accelerate wound healing. In addition, many other materials, such as metal-organic frameworks (MOFs) 152-154, metal nanozymes 155, 156, and probiotics 157 are used to mix with hydrogels to form composite materials that play a role in antibacterial and angiogenesis, or used for diabetic skin wounds. These materials have excellent performance and significant effects; however, they have not yet been applied to esophageal or gastric wounds. By selecting different types of nanomaterials and appropriate hydrogels, hybrid hydrogels with enhanced functionality can be obtained, achieving specific properties far beyond those of traditional hydrogels.

Metal-coordination hydrogels have immense potential for medical use 158. Some hydrogels based on Fe^3+^ exhibit extremely fast self-assembly (about 1 minute), a stronger hydrogel network, and a slower degradation rate, and show vigorous antibacterial activity against* E. coli* and* S. aureus* 159.

Nowadays, the intelligence of hydrogels is mainly reflected in pH response or temperature sensitivity, rather than in monitoring wound recovery. It is a new trend to combine hydrogel adhesives with capsule robots or fluorescence imaging technology to monitor wound healing and changes in gastric acid 160-167. Suppose the healing of gastric perforation can be monitored using hydrogel adhesives, it will not only effectively prevent secondary perforations but also be beneficial to the clinical research on gastric perforation healing. In addition, researchers continue to pursue good mechanical and hemostatic properties. Addressing the above limitations will effectively advance the research on hydrogel adhesives for clinical GI wound care.

Currently, some hydrogels used for hemostasis in the digestive tract have entered clinical trials. For example, UI-EWD, a new hemostatic powder, consists of aldehyde-glucan and succinic acid-modified ε-polyglucan 168. When it comes into contact with water, the powder will immediately transform into the high-adhesive hydrogel. The initial hemostasis rate of UI-EWD reached 94%, and only 19% of patients experienced rebleeding.

Integration with emerging technologies offers new opportunities for developing hydrogel adhesives. For example, 3D printing can design portable hydrogel preparations. Hydrogel structures for patients, targeted drug-delivery technologies can enhance localized treatment; imaging-guided hydrogels enable real-time monitoring of wound-healing progress. These technologies are critical to promoting the next generation of hydrogel adhesives in the upper GI field.

Biological safety is the key prerequisite for the application of hydrogel adhesives in upper GI wound management, and is mainly reflected in the following three aspects:

First, biocompatibility and mucosal tolerance should be ensured. Current studies mainly focus on short-term safety, while the risks associated with chronic exposure or repeated administration remain insufficiently studied. Therefore, it is necessary to assess the mucosal irritation, effects on epithelial barrier integrity, and the cytotoxicity and tissue compatibility of the hydrogel adhesives.

Second, degradation behavior and the toxicity of metabolic by-products require careful attention. There is a general lack of in-depth research on the correlation among their degradation kinetics, metabolic products and biosafety. Therefore, the degradation pathways of hydrogels and the mucosal toxicity and metabolic pathways of the degradation products should be elucidated.

Third, local immune responses and systemic toxicity must be considered. Since research on the chronic immune response, absorption, and accumulation risks of hydrogel adhesives remain limited, it is necessary to assess the local immune responses that hydrogels may induce, including macrophage polarization and activation of mucosal inflammatory pathways, and to reveal the correlation between the components of hydrogels and their immune activation.

Overall, biosafety evaluation of hydrogel adhesives is crucial for advancing their preclinical validation and translation into upper gastrointestinal tract therapies, and it also supports the development of safer, more controllable adhesive materials.

## 5. Conclusions

Upper GI disorders, including bleeding, ulceration, perforation, and postoperative defects, are very prevalent worldwide and seriously affect the quality of survival of most patients. Moreover, the acidic environment of the stomach makes gastric wound healing difficult. Hydrogel adhesives provide a unique combination of strong wet adhesion, acid resistance, mechanical compliance, injectability, and drug-loading capability, making them highly suitable for GI wound management. This review summarizes the significant progress of hydrogel adhesives across major clinical upper GI wounds, including hemostasis and ulcer sealing, perforation repair, assistance in ESD, and protection during radiotherapy via hydrogel spacers. We highlighted the key design principles and representative materials and therapeutic strategies. The challenges and perspectives of the hydrogel adhesives for upper GI wound healing are also discussed. Overall, hydrogel adhesives are promising candidates for upper GI wound treatment materials.

## Figures and Tables

**Table 1:** Hydrogel adhesives for gastric ulcers and gastric hemorrhages

Types of Hydrogels	Materials	Crosslinking Methods	Advantages	Refs.
CS-NAC/SA/TCP	CS-NAC SA TCP	Electrostatic interaction Hydrogen bonds Ionic crosslink	pH responsiveness Anti-inflammatory	19
PEI/PAA/CS	PEI PAA CS	Electrostatic interaction Hydrogen bonds	Ultrafast gelation (<3 s) Excellent tissue adhesion Gastric fluid resistance Hemostasis	53
CMCS/Lap/PVA	CMCS PVA Lap	Electrostatic interaction Hydrogen bonds	Injectability Antibacterial properties Hemostasis	60
HP powders	HTCC PA	Electrostatic interaction	Fast hemostatic capability Strong acid resistance	61
HA-CAT-NCSN	HA-Cat HA-NCSN	Thiourea-catechol coupling	Ultrafast gelation *In situ* gelation	62
HACN-PEG	HA-Cat HA-NCSN PEG	Thiourea-catechol coupling Disulfide bonds	Ultrafast gelation *In situ* gelation Hemostasis	29
GelMAc/PVAMA/MPDA@Cur	GelMAc PVAMA MPDA NPs Cur	π-π stacking Hydrogen bonds	Anti-inflammatory Stable under acidic/oxidative stress Promotes macrophage polarization and tissue repair	69
ISFIHs	AA AA-NHS	Covalent crosslink	Injectability pH-responsiveness	70
PU/SIS	PU SIS	Ionic bonds	pH-responsiveness	71
Oral keratin hydrogel	Keratins	Disulfide bonds	No endoscopic guidance	72
e-GLUE	SA PAM Cations	Electrostatic interaction	Strong electroadhesive	73
GastroShield	oxidized dextran PluPEI	Schiff base bonds	Sprayable Ultrafast gelation Strong tissue adhesion	12
MA-HA/AA10/Rg1	MA-HA AA ginsenoside Rg1	Photocrosslinking	Anti-inflammatory Hemostasis pH responsiveness	30

CS-NAC: N-acetylcysteine grafted chitosan; TCP: tilapia collagen peptide; PEI: polyethyleneimine; PAA: polyacrylic acid; CS: chitosan; CMCS: carboxymethyl chitosan; PVA: poly(vinyl alcohol); Lap: lithium magnesium sodium salt; HA-Cat: hyaluronic acid- catechol; HA-NCSN: hyaluronic acid-thiourea; PEG: polyethylene glycol; GelMAc: catechol motif-modified methacrylated gelatin; PVAMA: methacrylated poly (vinyl alcohol); MPDA NPs: mesoporous polydopamine nanoparticles; Cur: curcumin; AA: acryloyl-6-aminocaproic acid; AA-NHS: AA-g-N-hydroxysuccinimid; PU: Polyurethane; BIS: N,N'-methylenebisacrylamide; SIS: small intestinal submucosa; PAM: poly-acrylamide; PluPEI: amine-modified block-copolymer; MA-HA: methacryloylhaluronic acid.

**Table 2:** Hydrogel adhesives for gastric perforation

Types of Hydrogels	Materials	Crosslinking Methods	Advantages	Refs.
PEI/PAA powder	PEI PAA	Electrostatic interaction; Hydrogen bonds	Ultrafast self-gelling (< 2 s) *In situ* gelation Absorb interfacial water	81
CPO powder	CMCS γ-PGA OD	Amide bonds; Schiff base bonds	Fast self-gelling *In situ* gelation Absorb interfacial water Hemostasis Antibacterial properties	82
ABA triblock copolymer	PEG PNI-PAM A6ACA	Hydrophobic interactions; Hydrogen bonds	Rapid self-healing Injectability	83
DGL	GelMA Laponite nanoclay DNA	Electrostatic interaction; Covalent bonds	Dynamic reversibility Low swelling Wet adhesive properties Injectability Hemostasis	21
PTH	THMA gelatin collagenase type II APS	TG crosslinking of gelatin; Covalent bonds	*In situ* gelation Injectability	84
PTOPT	N-(2-aminoethyl)-N-[3-oxo-3-(phenethylamino)propyl]acrylamide	Cation-π interactions; π-π stacking	Injectability Self-healing Antibacterial properties	85
AN@CD-PEG&TQ	PEG-SC AN@CD nanoprobe anti-microbial peptide Tet213 pro-angiogenic peptide QK	Host/guest complexation; Amide bonds	Injectability Antibacterial properties Facilitating angiogenesis	86
GelMA-TA	GelMA TA	Hydrogen bonds	Anti-tensile healing Gastric fluid resistance	87
MCH	DMA Ca^2+^ SA VF AFGF	Covalent bonds; Ionic crosslink	Effective closure Easy operation Noninvasive delivery	88
EHO	CA-CS PAM	Photocrosslinking	Customizable Endoscopy delivery	89
hm-ApGltn MPs	Hydrophobically-modified ApGltn	Hydrophobic interactions	Anti-inflammatory Anti-fibrotic	91
hMPs	Hydrophobically-modified ApGltn	Hydrophobic interactions	Strong burst pressure Stable adhesion Sprayable	32
PSA	SF PAA	Hydrogen bonds; Electrostatic interactions; Chain entanglement derived from SF	Reliable adhesion via silk fibroin chain entanglement Strong wet adhesion by rapid dehydration effect	33
Janus hydrogel	PAASP Fe^3+^	Hydrogen bonds	Asymmetric adhesion Excellent stretchability Fatigue resistance Strong adhesion to bio-tissues Hemostasis Anti-postoperative tissue adhesion	77
Janus hydrogel	PAGG COS	Hydrogen bonds; Electrostatic interactions	Asymmetric adhesion Tissue adhesion Replacement of surgical suture Anti-postoperative tissue adhesion	96
Janus hydrogel	Catechol-grafted HA	Photocrosslinking	Asymmetric adhesion Anti-postoperative tissue adhesion Injectability	97
HADP	HA DA PBA	Boronate ester bonds; Photocrosslinking	Asymmetric adhesion Sprayable Anti-postoperative tissue adhesion	31
Acrylic acid/chitosan/tannic acid/Al^3+^	AA CS TA Al^3+^ SA Ca^2+^	Hydrogen bonds	Asymmetric adhesion High adhesive strength Anti-postoperative tissue adhesion	98
DQA	PDA QCS Acrylic acid ADM	Electrostatic interactions; Hydrogen bonds	Asymmetric adhesion Anti-postoperative tissue adhesion Antibacterial properties High burst pressure Replacement of surgical suture	99
PAM/TA	PAM SA TA	Hydrogen bonds	Asymmetric adhesion Anti-inflammatory	100
PAM-SA-NHA	PAM SA NHA	Covalent crosslink; Ionic crosslink	Low swelling properties Excellent mechanical and adhesion properties	103
ATGel	Poly(HEMA-NVP) Poly(AA-NHSAE)	Covalent crosslink	Gastric fluid resistance Instant and tough adhesion to gastric tissues Long-term adhesion	13

PEI: polyethyleneimine; PAA: polyacrylic acid; CMCS: carboxymethyl chitosan; γ-PGA: poly-γ-glutamic acid; OD: oxidized dextran; PEG: polyethylene glycol; PNI-PAM: poly(N-isopropylacrylamide); A6ACA : acryloyl-6-aminocaproic acid; GelMA: methacrylate gelatin; DNA: deoxyribonucleic acid; THMA: N-[Tris(hydroxymethyl)methyl]acrylamide; APS: ammonium persulfate; TA: tannic acid; EHO: endoscopy-deliverable hydrogel occlude; CA-CS: caffeic acid-grafted chitosan; DMA: dimethylacrylamide; CBA: N,N′-cystaminebis(acrylamide); SA: sodium alginate; VF: vonoprazan fumarate; AFGF: acidic fibroblast growth factor; ApGltn: Alaska pollock gelatin; SF: silk fibroin; PAASP: Polymeric N-acryloyl aspartic acid; PAGG: poly(N-acryloyl 2-glycine); COS: chitooligosaccharide; HA: hyaluronic acid; DA: dopamine; AA: acrylic acid; CS: chitosan; PDA: polydopamine; QCS: quaternary ammonium chitosan; PBA: phenylboronic acid; ADM: acellular dermal matrix; PAM: poly-acrylamide; NHA: nano-hydroxyapatite; HEMA-NVP: 2-hydroxyethyl methacrylate-co-N-vinylpyrrolidone; AA-NHSAE: acrylic acid-co-N-hydroxysuccinimide acrylate ester.

**Table 3:** Submucosal injection hydrogels for tissue lifting and indirect wound healing support

Types of Hydrogels	Materials	Crosslinking Methods	Advantages	Treatment	Refs.
CS/GP/Col	CS GP Col	Hydrogen bonds	Temperature sensitivity Hemostasis	Hemostasis	118
CS/GP/HPC/Col	CS GP Col HPC	Hydrogen bonds	Temperature sensitivity Stable adhesion	Hemostasis	120
CSLA/CS/GP	CSLA CS GP	Hydrogen bonds	Temperature sensitivity Injectability	Promoting wound healing	121
HpHCS-PVP-GP	HpHCS PVP GP	Hydrogen bonds	Temperature sensitivity Injectability High mechanical strength	Non	125
FS	SA F-127	Hydrogen bonds	Temperature sensitivity Injectability	Antibacterial Promoting wound healing	126
Az-CH-LA	Az-CH-LA	Photocrosslinking	Crosslinking through ultraviolet irradiation Hemostasis	Non	128
PSS/F127	Polystyrene sulfonate F-127	Hydrogen bonds	Conductive thermosensitive	Reducing tissue burns and bleeding	127
G-OALG	Gelatin OSA	Schiff base bonds	Controllable gelation viscosity and degradation rate Low cost	Non	113
GGH	GGH	Double-helix aggregation	Easy endoscopic injectability Good hemostatic property	Hemostasis	131
ISAHs	OSA SA-GSH	Hemi-thioacetal bonds	Gel stability Self-healing properties Injectability	Non	132
Dipeptide self-assembled hydrogels	FL YL LL YA	π-π stacking Hydrophobic effect	Self-healing properties Injectability	Non	133
CMS/Lap	CMS Lap	Electrostatic interaction	Self-healing properties Injectability	Hemostasis	134
CCS@Ag	Catechol Chitosan Ag	Schiff base bonds	Self-healing properties Injectability Antibacterial properties	Antibacterial	135
HBC-SA	HBC SA	Hydrogen bonds Hydrophobic interaction	Temperature sensitivity Injectability	Hemostasis	141
βCP-TET-ISO	β-CD PEGMA	Radical polymerization	Self-healing properties Injectability	Hemostasis Antibacterial Drug delivery	136
PLGA-*b*-PEG-*b*-PLGA	PLGA PEG	Block blend	Temperature sensitivity Injectability	Non	137
EISH	SA Laponite	Electrostatic interaction	Self-healing properties Injectability	Non	138
PGSH	PGS	Self-assembly	Self-healing properties Injectability Hemostasis	Hemostasis	139
Two-component *in-situ* hydrogel	CMCS SA	Schiff base bonds Thioether bonds	Injectability Hemostasis	Hemostasis Promoting wound healing	140

CS: chitosan; GP: β-glycerophosphate; Col: collagen; HPC: hydroxypropyl cellulose; CSLA: lactobionic acid-modified chitosan; HpHCS: chitosan solution with high pH value; PVP: polyvinylpyrrolidone; SA: sodium alginate; F-127: polyethylene-polypropylene glycol; GGH: gellan gum hydrogel; OSA: oxidized sodium alginate; SA-GSH: glutathione reduced-modified sodium alginate; FL: 9-fluorenylmethoxycarbonyl (Fmoc)-conjugated phenylalanine-leucine; YL: tyrosine-leucine; LL: leucine-leucine; YA: tyrosine-alanine; CMS: carboxymethyl starch; Lap: Laponite; HBC: hydroxybutyl chitosan.

**Table 4:** Key performance parameters of hydrogel adhesives for different upper gastrointestinal wound healing models

Applications	Design and synthesis	Key performance parameters					Refs.
		Gelation time	Adhesion strength	Acid resistance time	Hemostasis time	Others	
Gastric bleeding and ulcer	CS-NAC/SA/TCP	*In situ* gelation		> 2 h		Degradation rate: 39.51 ± 2.01% (pH 1.2, 3 days)	19
	PEI/PAA/CS	< 3 s	< 15.7 kPa (porcine stomach tissue after 1 h in acidic buffer)	28 days	33 s (rat liver model), 86 s (rabbit gastric artery model), 38.5 s (rabbit liver model)	Degradation rate: 46.4% (pH 1, 28 days), 35.4% (pH 4, 28 days)	53
	CMCS/Lap/PVA	4.69 ± 1.01 min	3.90 ± 0.25 kPa	14 days	< 90 s (rat liver model)	Swelling rate: 296.7 ± 142.5% Degradation rate: < 70%	60
	HP	< 5 s	< 3.6 kPa (porcine stomach at pH 1, HTCC/PA = 3:1)	10 days	208.3 s (rat tail bleeding model), 142.0 s (rat liver injury model)	Degradation rate: < 64.7% (10 days)	61
	HACN-PEG	1.7 s	< 10.4 kPa (chicken skin)	Overnight	59 ± 7.94 s (rat femoral artery model), < 2 min (porcine upper GI hemorrhage model)	Swelling rate: < 189%	29
	ISFIHs	< 9 min	6.63 kPa (porcine stomach tissue)		Immediate hemostasis	Swelling rate: 430%-670% (pH 7.4), 30%-90% (pH 2.0) Adhesion duration: 28 days	70
	e-GLUE	40 - 80 s		30 days	Immediate hemostasis	Adhesion duration: 4-8 days (colon)	73
	GastroShield	2 - 17 s		≥ 1 week (pH 2)	Immediate hemostasis	Adhesion duration: 7 days Swelling rate: ~ 50% Degradation rate: < 50% (pH 2, 7 days)	12
Gastric perforation repair	PEI/PAA powder	< 2 s	74.6 kPa (wet chicken skin)	≥ 2 weeks		Adhesion duration: ≥ 30 days	81
	CPO powder	14.7 ± 3.1 s	55.4 ± 4.5 kPa (wet porcine skin)		36.7 ± 4.5 s	Swelling rate: 1530%-1840% Degradation rate: 78.9 ± 3.9%	82
	PTOPT		15.3 kPa (stomach), 20.5 kPa (skin)	60 h	Immediate hemostasis	Adhesion duration: 10 days Degradation rate: 83.28% (pH 2.5)	85
	MCH		81 ± 5 kPa (glass)		< 10 s (≈ 5 s) (rabbit liver hemorrhage model)	Adhesion duration: ≥ 24 h Swelling rate: 3500%	88
	EHO			≥ 24 h (pH 2.0)		Swelling rate: 500% (pH 7.4), 580% (pH 2.0)	89
	PAASP/Fe^3+^ Janus hydrogel	20 min	The adhesive side: 106 kPa (porcine skin), the non-adhesive side: 7.9 kPa	≥ 6 h	≤ 10 s	Swelling rate: ~120% (pH 3)	77
	HAD Janus hydrogel	< 3 s	~8.5 kPa (porcine stomach)	≥ 6 h	Immediate hemostasis	Swelling rate: 400% (pH 1.2)	97
	HADP	Within seconds		≥ 6 h		Swelling rate: 171 ± 17% (pH 7.4, 2 days) Degradation rate: 90.8 ± 10.3% (pH 7.4, 7 days)	31
	DQA	≤ 1 h	59.1 kPa (porcine skin), 30.4 kPa (stomach tissue), 23.7 kPa (intestine tissue)	14 days		Adhesion duration: ≥ 14 days (pH 1.21) Swelling rate: 22.8 ± 4.5% (pH 1.21, 14 days), 48.1 ± 9.1% (pH 1.21, 14 days)	99
	PAM-SA-NHA	5 s	108 J/m² (porcine skin)		Immediate hemostasis	Adhesion duration: ≥ 12 h	103
	ATGel	Within seconds	40 - 60 kPa	10 days	Immediate hemostasis	Adhesion duration: ≥ 240 h (pH 2.0) Swelling rate: 82 % (pH 2.0, 24 h)	13

## Abbreviations

GI: gastrointestinal
3D: three-dimensional
CMCS: carboxymethyl chitosan
SA: sodium alginate
ODE: oxidized dextran
DNA: deoxyribonucleic acid
RNA: ribonucleic acid
EC: esophageal cancer
ESD: endoscopic submucosal dissection
EV: extracellular vesicles
GIB: gastrointestinal bleeding
CS: chitosan
CS-NAC: N-acetylcysteine grafted chitosan
TCP: tilapia collagen peptide
PEI: polyethyleneimine
PAA: polyacrylic acid
Lap: lithium magnesium sodium salt
PVA: poly(vinyl alcohol)
HA-Cat: hyaluronic acid-catechol
HA-NCSN: hyaluronic acid-thiourea
PEG: polyethylene glycol
PVAMA: methacrylated poly(vinyl alcohol)
GelMAc: catechol motif-modified methacrylated gelatin
MPDA NPs: mesoporous polydopamine nanoparticles
Cur: curcumin
AA-NHS: AA-g-N-hydroxysuccinimid
PU: Polyurethane
BIS: N,N′-methylenebisacrylamide
SIS: small intestinal submucosa
PluPEI: amine-modified block-copolymer
γ-PGA: poly-γ-glutamic acid
OD: oxidized dextran
PNI-PAM: poly(N-isopropylacrylamide)
A6ACA: acryloyl-6-aminocaproic acid
GelMA: methacrylate gelatin
TA: tannic acid
DMA: dimethylacrylamide
CBA: N,N′-cystaminebis(acrylamide)
AFGF: acidic fibroblast growth factor
VF: vonoprazan fumarate
SF: silk fibroin
PAASP: Polymeric N-acryloyl aspartic acid
HA: hyaluronic acid
DA: dopamine
AA: acrylic acid
PDA: polydopamine
QCS: quaternary ammonium chitosan
PBA: phenylboronic acid
ADM: acellular dermal matrix
PAM: poly-acrylamide
NHA: nano-hydroxyapatite
HPC: hydroxypropyl cellulose
Col: collagen
GP: β-glycerophosphate
CSLA: lactobionic acid-modified chitosan
HpHCS: chitosan solution with high pH value
PVP: polyvinylpyrrolidone
F-127: polyethylene-polypropylene glycol
GGH: gellan gum hydrogel
OSA: oxidized sodium alginate
SA-GSH: glutathione reduced-modified sodium alginate
FL: 9-fluorenylmethoxycarbonyl (Fmoc)-conjugated phenylalanine-leucine
YA: tyrosine-alanine
LL: leucine-leucine
YL: tyrosine-leucine
CMS: carboxymethyl starch
HBC: hydroxybutyl chitosan
OARs: organs at risk
COFs: covalent organic frameworks
OCNTs: oxidized carbon nanotubes
POD: peroxidase
CAT: catalase
MOF: metal-organic framework
